# Analysis of Antioxidant Constituents from *Ilex rotunda* and Evaluation of Their Blood–Brain Barrier Permeability

**DOI:** 10.3390/antiox11101989

**Published:** 2022-10-06

**Authors:** Chang-Kwon Kim, Jeongjun Ahn, Jayeon Yu, DucDat Le, Sanghee Han, Mina Lee

**Affiliations:** College of Pharmacy and Research Institute of Life and Pharmaceutical Sciences, Sunchon National University, 255 Jungangno, Suncheon 57922, Korea

**Keywords:** *Ilex rotunda*, free radical scavenging activity, HPLC-DPPH, validation method, antioxidant, PAMPA-BBB

## Abstract

*Ilex rotunda* Thunb., has been used to treat common cold, tonsillitis, and eczema. It is also a source of antioxidants. However, information regarding its antioxidative phytochemical composition is still incomplete and limited. In this present study, we initially determined DPPH radical scavenging activity of the extracts of *I. rotunda* fruits, twigs, and leaves. Among them, the twig extract exhibited a potential of antioxidant capacity. Based on antioxidant effect guided experiments, extraction condition using 80% EtOH was then optimized. DPPH and ABTS radical scavenging assays were also performed for fractions. The *n*-butanol fraction showed the highest antioxidant effect. Using chromatographic methods, eight marker compounds (**1**–**8**) were further isolated. Their structures were determined by spectroscopic and mass data. Method validation was employed to quantitate contents of these eight marker compounds. Subsequently, the HPLC-DPPH method was used to evaluate the contribution of certain compounds to total antioxidant activity of the extract. Lastly, parallel artificial membrane permeability assay for blood–brain barrier (PAMPA-BBB) was applied to investigate brain-penetrable antioxidants from *I. rotunda* extract. As a result, compound **7** (4,5-dicaffeoylquinic acid) showed significant antioxidant activity and penetration across the BBB via transcellular passive diffusion. Our findings suggested that compound **7** can be used as a therapeutic potential candidate in natural product-based central nervous system (CNS) drug discovery.

## 1. Introduction

Oxidative stress occurs when there is an imbalance between free radicals and antioxidants in the body [1]. The body’s cells produce free radicals during normal metabolic processes. They also produce antioxidants that can neutralize these free radicals [2]. In general, the body can maintain a balance between antioxidants and free radicals [3]. However, when there are more free radicals than antioxidants, free radicals can damage fatty tissue, DNA, and proteins in the body [4]. Several factors contribute to oxidative stress and excess free radical production [5]. These factors include cigarette smoking, metabolized alcohol and drugs, certain pesticides and cleaners, and environmental factors such as pollution and radiation [6,7].

Oxidative stress can lead to various diseases, including inflammation, aging, cancer, diabetes, cardiovascular, and hypertension [8,9,10]. It could also contribute to several neurodegenerative conditions such as Alzheimer’s and Parkinson’s diseases [11]. The brain is particularly vulnerable to oxidative stress because brain cells require a substantial amount of oxygen [12]. During oxidative stress, excess free radicals in the central nervous system (CNS) can damage structures inside brain cells and modify amyloid-beta peptides, which may increase the risk of neurodegenerative diseases (NDs) [13]. Thus, therapeutic strategies for preventing free radicals are widely recognized. Considerable efforts are currently dedicated to the development of antioxidants as neuroprotective drugs.

*Ilex rotunda* belongs to genus *Ilex* of Aquifoliaceae family. This plant is distributed in the east Asia region including China, Japan, Taiwan, and Korea. Previous studies have reported that this plant contains triterpenes and their saponins, sesquiterpenes, hemiterpene glycosides, flavonoid glycosides, and aromatic compounds [14]. Modern pharmacological studies have shown that *I. rotunda* has cardiovascular system-protecting, colitis-associated cancer (CAC)-preventing, anti-inflammatory, antibacterial, and antioxidative effects [15,16]. However, the antioxidative properties of chemical constituents derived from this plant have not been studied well in vitro.

Therefore, the present study reports approaches of searching for antioxidant constituents in *I. rotunda* by 1,1-diphenyl-β-picrylhydrazine (DPPH) and 3-ethylbenzothiazoline-6-sulfonic acid diammonium salt (ABTS) radical scavenging assays. These eight marker compounds isolated from an active fraction of *I. rotunda* extract were validated using the established method, and antioxidant constituents were rapidly identified through the high-performance liquid chromatography-1,1-diphenyl-β-picrylhydrazine (HPLC-DPPH) method [17]. Finally, a parallel artificial membrane permeability assay for blood–brain barrier (PAMPA-BBB) was applied to assess brain-penetrable antioxidants from *I. rotunda* extract [18].

## 2. Materials and Methods

### 2.1. Plant Materials

*I. rotunda* fruits, twigs, and leaves were collected from Suncheon, Korea, in October 2020. The plant was identified and authenticated by Prof. Mina Lee (College of Pharmacy, Sunchon National University). A voucher specimen (SCNUP-27) was deposited in the laboratory of Pharmacognosy, College of Pharmacy, Sunchon National University (Suncheon, Korea).

### 2.2. Preparation of Extracts

For radical scavenging assay, samples of *I. rotunda* fruits, twigs, leaves (1 g, each) were dried, ground, and then extracted three times with 80% ethanol (20% water) using ultrasonication at room temperature (12 min × 2 cycles), respectively. Extracts were concentrated in vacuum at 39 °C. To prepare ethanol extracts, 1 g of ground *I. rotunda* twigs was mixed with 10 mL of 0, 20, 40, 60, 80, or 100% EtOH and extracted using ultrasonication at room temperature (120 min × 2 cycles), respectively. Extracts were filtered through No. 2 Whatman filter paper (Whatman, Pleasanton, CA, USA) and evaporated in vacuum at 39 °C using a rotary evaporator (Eyela, Tokyo, Japan). Finally, concentrated extracts were kept in the dark at 4 °C. 

### 2.3. DPPH Radical Scavenging Assay

The radical scavenging effect of 2,2-diphenyl-1-picrylhydrazyl (DPPH; Thermo Fisher Scientific, Ward Hill, MA, USA) was measured using our previous method [19]. Briefly, 0.1 mL of each sample solution (dissolved in EtOH) was mixed with 0.1 mL of 0.2 mM DPPH and allowed to stand at RT for 30 min under shade. The absorbance at 517 nm was measured using a microplate spectrophotometer (Epoch, Biotek Instruments, Inc., Winooski, VT, USA). Ascorbic acid (100 μg/mL) (Sigma–Aldrich, Co., St. Louis, MO, USA) was used as a positive control. The percentage of DPPH reduction between the treated sample and negative control well was calculated with the following formula: %EC = (A control − A sample) * 100/(A control), where A sample was the absorbance of the sample and A control was the absorbance of untreated sample. Results are indicated as EC_50_, which correspond to the sample concentration (μg/mL) required to inhibition by 50% of the initial DPPH radical scavenging activity under the given experimental conditions.

### 2.4. ABTS Radical Scavenging Assay

ABTS radical inhibitory activity was measured by mixing 100 µL of each sample solution (dissolved in EtOH) and 100 µL of ABTS solution (7 mM 2,2′-azino-bis (3-ethylbenzothiazoline-6-sulfonic acid diammonium salt, ABTS, Sigma–Aldrich, Co., St. Louis, MO, USA) mixed with 2.45 mM potassium persulfate). After incubating at RT for 6 min, absorbance of the mixture was measured at 734 nm. Ascorbic acid (100 µg/mL) was used as the positive control: %EC = (A control − A sample) * 100/(A control), where A sample was absorbance of the sample and A control was absorbance of the untreated sample [20].

### 2.5. Fractionation and Separation of Marker Compounds **1**–**8**

Dried *I. rotunda* twigs (3.0 kg) were extracted with 80% ethanol by sonication at room temperature (2 h × 4 cycles). Extract was dried with a final weight of 37.2 g. This total extract was then suspended in H_2_O and partitioned in a regular sequence with *n*-hexane, CH_2_Cl_2_, EtOAc, and *n*-butanol to obtain 2.7 g, 1.9 g, 6.2 g, and 10.8 g residues, respectively. Among them, the *n*-butanol fraction exhibited potent antioxidant activities in DPPH and ABTS radical scavenging assays. Thus, this fraction was separated by preparative reversed-phase HPLC using a Triart C_18_ column (20 mm × 250 mm, 5 μm, YMC, Tokyo, Japan) at 9.0 mL/min with CH_3_CN-H_2_O gradient (10:90–100:0) and detection wavelength of (λ_max_) 254 nm, yielding 40 peaks rich in secondary metabolites. Compounds **1**, **2**, and **4** (*t*_R_ 18.2, 23.1, and 25.4 min) were obtained from subfractions 18, 23, and 25, respectively. The subfraction 24 was further isolated by semipreparative HPLC on a Triart C_18_ column (10 mm × 250 mm, 5 μm, YMC, Tokyo, Japan) at 3.0 mL/min with CH_3_CN-H_2_O isocratic (25:75) and detection wavelength of 254 nm, yielding compound **3** (*t*_R_ 72.3 min). Reference standard of **3** was additionally purchased from Sigma–Aldrich (Burlington, MA, USA) for validation study. Purification of subfraction 26 was accomplished by semipreparative HPLC on a Triart C_18_ column (10 mm × 250 mm, 5 μm, YMC, Tokyo, Japan) at 3.0 mL/min with CH_3_CN-H_2_O gradient (10:90–25:75) and detection wavelength of 254 nm to yield compound **5** (*t*_R_ 12.1 min). Subfraction 27 was separated by semipreparative HPLC on a Triart C_18_ column (10 mm × 250 mm, 5 μm, YMC, Tokyo, Japan) at 3.0 mL/min with CH_3_CN-H_2_O isocratic (25:75) and detection wavelength of 254 nm, yielding compounds **6** and **7** (*t*_R_ 21.3 and 23.4 min), respectively. Lastly, subfraction 36 was further isolated by semipreparative HPLC on a Triart C_18_ column (10 mm × 250 mm, 5 μm, YMC, Tokyo, Japan) at 3.0 mL/min with CH_3_CN-H_2_O isocratic (30:70) and detection wavelength of 254 nm, yielding compound **8** (*t*_R_ 48.7 min).

### 2.6. Method Validation

#### 2.6.1. Detection Wavelength

Compound **1**, phenolic glycoside, indicated UV absorption maxima at 213 and 254 nm. Compounds **2** and **4**–**8**, mono-, di-, tri-caffeoylquinic acids (CQAs), showed UV absorption maxima at 204, 216, and 326 nm. Compound **3**, flavonoid glycoside, displayed UV absorption maxima at 204, 254, and 353 nm. Compound **4**, hemiterpene glycoside, displayed UV absorption maxima at 192 and 326 nm. Therefore, UV wavelengths were collected at 254 and 326 nm for detection of two compounds (**1** and **3**) and six compounds (**2** and **4**–**8**), respectively (Figure S1).

#### 2.6.2. Preparation of Calibration Standard Solution

The eight marker compounds **1**–**8** reached purities over 96.22% based on the detection of their signals with the high-performance liquid chromatography-photodiode array (HPLC-PDA) system. Standard stock solution was prepared at a concentration of 1000 μg/mL. It was then diluted by adding MeOH to prepare working concentrations. The solution was sealed by elastic plastic film and stored in a refrigerator at 4 °C for analysis. Calibration curves were built using six different concentrations for each analyte. In detail, different concentrations ranging from 6.25 to 200 μg/mL for compounds **1** and **3**; 12.5 to 400 μg/mL for compounds **2**, **4**, **5**, and **8**; and 25 to 800 μg/mL for compounds **6** and **7** were prepared. Linearity of calibration curves was determined by plotting the mean peak area (y axis) versus concentration (x axis) for each analyte in that range. The limit of detection (LOD) and limit of quantification (LOQ) were calculated as follows: LOD = 3.3 × SD/S and LOQ = 10 × SD/S, respectively, where SD was the standard deviation and S was the slope of the calibration curve. Intra- and interday variabilities of the *I. rotunda* extract were evaluated for each sample with six replicates during a day and by duplicating experiments on six consecutive days, respectively. Relative standard deviation (RSD) was calculated to evaluate precision using the following equation: RSD (%) = SD × 100/mean measured concentration. To verify the accuracy, a recovery test was performed using spiked *I. rotunda* samples at three different concentrations (low, medium, high): compounds **1** and **3** (200, 100, 40 μg/mL); compounds **2**, **4**–**5**, and **8** (400, 160, 64 μg/mL); and compounds **6** and **7** (800, 320, 128 μg/mL). The mean recovery (%) was calculated using the following equation: recovery (%) = detected concentration × 100/(original concentration + spiked concentration).

#### 2.6.3. Chromatographic and Separation Conditions

Chemical profiling of *I. rotunda* with qualification and validation of eight marker compounds was performed with an HPLC (Waters, Houston, TX, USA) equipped with an autosampler, a degasser, a quaternary solvent pump, and photodiode array (PDA) detector at 25 °C. Eight marker compounds were analyzed using a Triart C_18_ column (4.6 × 250 mm, 5 μm, YMC, Tokyo, Japan) at 35 °C with a flow rate of 0.8 mL/min and an injection volume of 5 μL. The detection was performed with an ultraviolet (UV) detector at a wavelength of 254 nm. The mobile phase consisted of a solvent system of phase A (water containing 0.1% formic acid) and phase B (acetonitrile) with gradient elution as follows: 10–10% (B) from 0 to 2 min, 10–15% (B) from 2 to 15 min, 15–22% (B) from 15 to 17 min, 22–22% (B) from 17 to 25 min, 22–30% (B) from 25 to 30 min, 30–50% (B) from 30 to 35 min, 50–100% (B) from 35 to 40 min, 100–100% (B) from 40 to 45 min, 100–10% (B) from 45 to 47 min, 10–10% (B) from 47 to 50 min. The column was then re-equilibrated with 10% (B) until the end of analysis. The range of PDA detection wavelength was set at 200–400 nm. Of these, chromatographic data at 254 nm were recorded.

#### 2.6.4. Mass Conditions

The mass spectrometer was operated using a Waters Quattro Micro Mass^TM^ (Waters, Milford, MA, USA) equipped with an electrospray ionization (ESI) source. The instrument was operated in positive and negative ion modes. MS conditions were as follows: capillary voltage, 3.0 kV; cone voltage, 50 V; extractor voltage, 3 V; RF lens voltage, 0 V; source temperature, 100 °C; desolvation temperature, 300 °C; desolvation gas, 450 L/h and cone gas, 40 L/h. All data acquisition and process were performed using Empower 3 and Waters MassLynx 4.1 software. (Waters, MA, USA).

### 2.7. HPLC-DPPH Method and ELISA Assay

An amount of 100 μL of the *I. rotunda* extract (1.3 mg/mL) dissolved in methanol and 100 μL of the DPPH solution (1.2 mg/mL in methanol) were mixed and incubated for 30 min at 37 °C, protected from light. After that, the mixture was filtered through a 0.22 μm filter for HPLC analysis. The control sample was prepared by mixing 100 μL methanol with 100 μL of the extract. Both mixtures were analyzed using the same established analytical methods [17]. The outflow was monitored at 254 and 326 nm wavelength. Active compounds **2** and **6**–**8** were further experimented for their antioxidant activities using the above DPPH method on enzyme-linked immunosorbent assay (ELISA). The ELISA assay was performed by following procedure. A 100 μL of DPPH solution (0.2 mM) was added to 100 μL of the sample on a 96-well plate, mixed for 5 s, and reacted for 30 min under shade. The absorbance was measured at 517 nm using a microplate spectrophotometer (Epoch, Biotek Instruments, Inc., Winooski, VT, USA). Compounds **2** and **6**–**8** were prepared at concentrations ranging from 2.5 to 40 μM. Ascorbic acid (100 μg/mL) (Sigma–Aldrich, Co., St. Louis, MO, USA) was used as the positive control.

### 2.8. Parallel Artificial Membrane Permeability Assay for the Blood–Brain Barrier (PAMPA-BBB)

The PAMPA-BBB experiment was carried out according to the study by Könczöl et al. [21]. A slightly modified version of the PAMPA-BBB was used to assess effective permeability (P_e_, cm/s) of compounds of *I. rotunda* [22]. Briefly, 20 μL of stock solution of *I. rotunda* extract (10 mg/mL in MeOH) or test compound (10 mM in MeOH) was mixed with 180 μL of phosphate buffered saline (PBS, pH 7.4, 10 mM) to obtain the starting donor solution. Subsequently, the filter membrane of the donor (top) plate (96-well polycarbonate-based filter plate, Multiscreen-IP, MAIPTR10, pore size 0.45 μm, Milipore) was coated with 5 μL of porcine polar brain lipid extract (PBLE) solution (16.0 mg PBLE + 8.0 mg cholesterol dissolved in 600.0 μL *n*-dodecane). Then, 150.0 μL of the filtrate was placed on the membrane. The bottom (acceptor) plate (96-well microtiter plate, Multiscreen^®^, MATRNPS50, Milipore) was filled with 300.0 μL buffer solution (PBS, pH 7.4, 10 mM). The donor plate was carefully located on the acceptor plate to form a “sandwich”. It was incubated at 37 °C for 4 h without direct right exposure. After incubation, PAMPA plates were separated. Concentrations of identified compounds of *I. rotunda* in the starting donor solution and in acceptor and donor wells were determined in triplicate based on chromatographic peak areas derived from the same established analytical methods. Using these data, the effective BBB permeability (log *P*_e_) of each test compound was calculated using the previously reported equations [23].

$$P_{e}=\frac{-\ln\left[1-C_{A(t)}/C_{\mathrm{equilibrium}\;}\right]}{A\times \left(\frac{1}{V_{D}}\frac{1}{V_{A}}\right)\times t}$$

where *P*_e_ is permeability in cm s^−1^. *A* = effective filter area = *f* × 0.3 cm^2^; *V*_D_ = donor well volume = 150 μL; *V*_A_ = acceptor well volume = 300 μL; *t* = incubation time (s) = 14,400; *C*_A(*t*)_ = compound concentration in the acceptor well at time *t*; and *C*_D(*t*)_ = compound concentration in the donor well at time *t*. C_equilibrium_ is calculated as follows:

$$C_{\mathrm{equilibrium}}=\left[C_{D(t)}\times V_{D}+C_{A(t)}\times V_{A}\right]/\left(V_{D}+V_{A}\right)$$

### 2.9. Statistical Analysis

All acquisition data are represented as means ± standard deviations (S.D.) of at least three independent experiments. Nonparametric one-way ANOVA followed by Dunnett’s multiple comparison test was performed using Graphprism version 8.0.1 software (GraphPad Software, La Jolla, CA, USA). * *p* < 0.05, ** *p* < 0.01, and *** *p* < 0.001, compared to controls were accepted as statistically significant.

## 3. Results

### 3.1. Screened DPPH and ABTS Activities Guided Extraction and Solvent Selection

**Figure 1:**
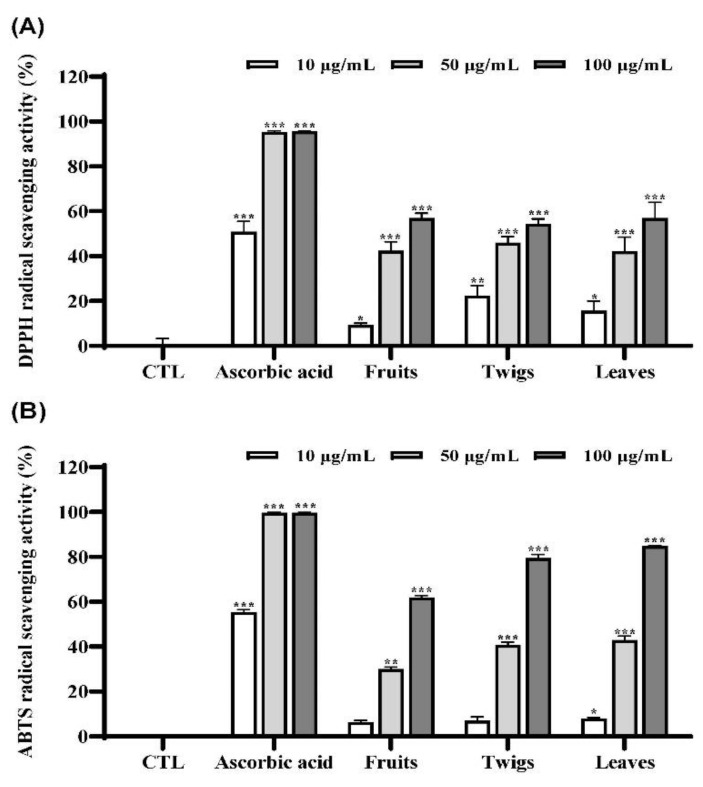
Effects of the extracts of *I. rotunda* fruits, twigs, and leaves on DPPH (**A**) and ABTS (**B**) radical scavenging assays. The data are expressed as the mean ± SD (*n* = 3) of three individual experiments. * *p* < 0.05, ** *p* < 0.01, and *** *p* < 0.001, compared with control (CTL) (blank sample).

DPPH and ABTS assays are simple tests that can give a first indication of radical scavenging potential of extracts of *I. rotunda* fruits, twigs, and leaves. Amounts of free DPPH and ABTS radicals were scavenged by tested samples and calculated with reference to the control (without sample addition). Ascorbic acid (100 μM) was used as a positive control. In the DPPH assay, the twig extract (45.9%) exhibited stronger radical scavenging activity than fruit (42.4%) and leaf (42.2%) extracts at 50 µg/mL (Figure 1A). In the ABTS assay, the twig (40.6%) extract showed similar strong activity compared to leaf (42.9%) extract at 50 µg/mL (Figure 1B). Obtained yield of twig (24.4%) was higher than that of leaf (21.0%). Thus, twig extract was selected as the material in our further research.

After the first experiments, DPPH and ABTS assays were also performed using *I. rotunda* twigs (extracted with 0%, 20%, 40%, 60%, 80%, and 100% EtOH) at different concentrations. The 80% EtOH twig extract showed a higher radical scavenging activity (69.0% inhibition) in DPPH assay than other twig extracts at the same concentration of 50 µg/mL (Figure S2A). In the ABTS assay, the 80% EtOH (71.9%) extract showed similar activity to the 20% EtOH (83.1%) extract at 50 µg/mL (Figure S2B). The obtained yield using 80% EtOH (24.4%) was higher than that using 20% EtOH (23.8%). Thus, 80% EtOH was an optimized extraction condition. 

### 3.2. Antioxidant Activities of Fractions

**Figure 2:**
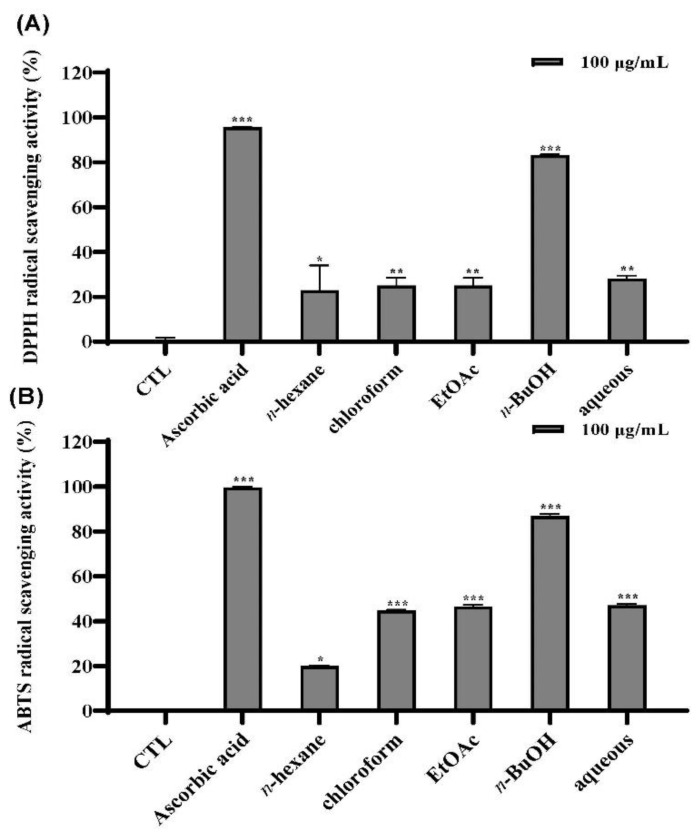
Effects of the fractions of *I. rotunda* twigs on DPPH (**A**) and ABTS (**B**) radical scavenging assays. The data are indicated as the mean ± SD (*n* = 3) of three individual experiments. * *p* < 0.05, ** *p* < 0.01, and *** *p* < 0.001, compared with control.

The 80% EtOH extract of *I. rotunda* twigs was successfully partitioned into *n*-hexane, CH_2_Cl_2_, EtOAc, *n*-BuOH, and aqueous fractions. Antioxidant activities of these fractions were also evaluated with DPPH and ABTS assays. Results revealed that the *n*-BuOH fraction showed the highest antioxidant activity in both DPPH and ABTS experiments. It showed 83.1% and 86.8% radical scavenging activities at 100 µg/mL in DPPH and ABTS assays, respectively (Figure 2A,B). Thus, chemical constituents of this fraction were extensively investigated. 

### 3.3. Isolation and Identification of Marker Compounds **1**–**8**

Spectroscopic Data of Compounds **1**–**8**

Syringin (**1**): White amorphous powder; ESI-MS: 395.01 [M + Na]^+^ (C_17_H_24_O_9_Na); ^1^H NMR (CD_3_OD, 400 MHz): δ 6.74 (2 H, s, H-3, and H-5), 6.54 (1 H, d, *J* = 15.8 Hz, H-7), 6.32 (1 H, dt, *J* = 15.8, 5.6 Hz, H-8), 4.88 (1 H, d, *J* =7.5 Hz, H-1’), 4.21 (2 H, d, *J* = 5.6 Hz, H-9), 3.85 (6 H, s, 2,6-OCH_3_), 3.20–3.79 (6 H, m, sugar H); ^13^C NMR (CD_3_OD, 100 MHz): δ 154.35 (C-2, C-6), 135.8 (C-7), 135.2 (C-1), 131.3 (C-4), 130.0 (C-8), 105.4 (C-3 and C-5), 105.3 (C-1’), 78.3 (C-5’), 77.8 (C-3’), 75.7 (C-2’), 71.3 (C-4’), 63.5 (C-9), 62.5 (C-6’), 57.0 (2,6-OCH_3_).

Chlorogenic acid (**2**): White amorphous powder; ESI-MS: 353.01 [M-H]^-^ (C_16_H_17_O_9_); ^1^H NMR (CD_3_OD, 400 MHz): δ 7.58 (1 H, d, *J* = 16 Hz, H-7′), 7.04 (1 H, d, *J* =2 Hz, H-2′), 6.93 (1 H, dd, *J* = 2, 8 Hz, H-6′), 6.77 (1 H, d, *J* = 8 Hz, H-5′), 6.26 (1 H, d, *J* = 16 Hz, H-8′), 5.34 (1 H, ddd, *J* = 3, 3, 4 Hz, H-3), 4.16 (1 H, ddd, *J* = 3, 9, 9 Hz, H-5), 3.63 (1 H, dd, *J* = 3, 9 Hz, H-4), 2.00–2.00 (4 H, overlap); ^13^C NMR (CD_3_OD, 100 MHz): δ 177.0 (C-7), 168.60 (C-9′), 149.4 (C-4′), 146.80 (C-7′), 146.79 (C-3′), 127.9 (C-1′), 122.9 (C-6′), 116.4 (C-5′), 115.8 (C-8′), 115.1 (C-2′), 76.1 (C-1), 73.4 (C-4), 71.9 (C-3), 71.3 (C-5), 38.7 (C-6), 38.1 (C-2).

Rutin (**3**): Yellow amorphous powder; ESI-MS: 609.05 [M-H]^-^ (C_27_H_29_O_16_); ^1^H NMR (CD_3_OD, 400 MHz): δ 7.66 (1 H, d, *J* = 2.0 Hz, H-2′), 7.63 (1 H, dd, *J* = 8.5, 2.0 Hz, H-6′), 6.87 (1 H, d, *J* = 8.5 Hz, H-5′), 6.39 (1 H, d, *J* = 2.0 Hz, H-8), 6.20 (1 H, d, *J* = 2,0 Hz, H-6), 5.11 (1 H, d, *J* =7.8 Hz, H-1′′), 4.51 (1 H, d, *J* = 1.8 Hz, H-1′′′), 3.80 (1 H, dt, *J* = 10.9, 1.0 Hz, Hb-6′′), 3.63 (1 H, dd, *J* = 3.5, 1.8 Hz, H-2′′′), 3.54 (1 H, dd, *J* = 9.5, 3.5 Hz, H-3′′′), 3.26–3.48 (4 H, m, H-2′′′, H-3′′, H-4′′, H-5′′), 3.44 (1 H, m, H-5′′′), 3.39 (1 H, m, Ha-6′′), 3.27 (1 H, m, H-4′′′), 1.12 (3 H, d, *J* = 6.1 Hz, C-6′′′); ^13^C NMR (CD_3_OD, 100 MHz): δ 179.4 (C-4), 166.1 (C-7), 163.0 (C-5), 159.3 (C-9), 158.5 (C-2), 149.8 (C-4′), 145.8 (C-3′), 135.6 (C-3), 123.5 (C-6′), 123.1 (C-1′), 117.7 (C-2′), 116.1 (C-5′), 105.6 (C-10), 104.7 (C-1′′), 102.4 (C-1′′′), 99.9 (C-6), 94.8 (C-8), 78.2 (C-5′′), 77.2 (C-3′′), 75.7 (C-2′′), 73.9 (C-4′′′), 72.2 (C-3′′′), 72.1 (C-2′′′), 71.4 (C-4′′), 69.7 (C-5′′′), 68.5 (C-6′′), 17.8 (C-6′′′).

Rotundarpenoside B (**4**): Yellow amorphous powder; ESI-MS: 425.06 [M-H]^-^ (C_20_H_25_O_10_); ^1^H NMR (CD_3_OD, 400 MHz): δ 7.53 (1 H, d, *J* = 15.6 Hz, H-7), 7.02 (1 H, d, *J* = 2.1 Hz, H-2), 6.92 (1 H, dd, *J* = 8.4, 2.1 Hz, H-6), 6.75 (1 H, d, *J* = 8.4 Hz, H-5), 6.25 (1 H, d, *J* = 15.6 Hz, H-8), 5.67 (1 H, m, H-2′), 4.57 (2 H, br s, H-4′), 4.41 (1 H, dd, *J* = 11.4, 5.8 Hz, H-1′a), 4.27 (1 H, d, *J* = 7.4 Hz, H-1′′), 4.24 (1 H, d, *J* = 5.8 Hz, H-1′b), 3.85–3.17 (6 H, m, sugar H); ^13^C NMR (CD_3_OD, 100 MHz): δ 168.8 (C-9), 149.5 (C-3), 147.2 (C-4), 146.7 (C-7), 135.9 (C-3′), 127.6 (C-1), 124.9 (C-6), 123.0 (C-2′), 116.5 (C-5), 115.1 (C-2), 114.8 (C-8), 103.1 (C-1′′), 78.0 (C-3′′), 77.9 (C-5′′), 74.9 (C-2′′), 71.5 (C-4′′), 69.6 (C-4′), 66.0 (C-1′), 62.7 (C-6′′), 14.2 (C-5′).

3,4-Dicaffeoylquinic acid (**5**): Yellow amorphous powder; ESI-MS: 515.05 [M-H]^-^ (C_25_H_23_O_12_); ^1^H NMR (CD_3_OD, 400 MHz): *δ* 7.57 (1 H, d, *J* = 15.6 Hz, H-7’), 7.52 (1 H, d, *J* = 15.6 Hz, H-7’’), 7.02 (1 H, s, H-2’), 7.01 (1 H, s, H-2’’), 6.90 (1 H, dd, *J* = 7.8, 1.8 Hz, H-6’), 6.88 (1 H, dd, *J* = 7.8, 1.8 Hz, H-6’’), 6.74 (1 H, d, *J* = 7.8 Hz, H-5’), 6.72 (1 H, d, *J* = 7.8 Hz, H-5’’), 6.26 (1 H, d, *J* = 15.6 Hz, H-8’), 6.25 (1 H, d, *J* = 15.6 Hz, H-8’’), 5.62 (1 H, m, H-3), 5.07 (1 H, m, H-5), 4.26 (1 H, m, H-4), 2.34 (2 H in total, m, H-2), 2.13 (2 H in total, m, H-6); ^13^C NMR (CD_3_OD, 100 MHz): *δ* 178.2 (C-7), 168.5 (C-9’), 168.4 (C-9’’), 149.6 (C-3’, 3’’), 147.3 (C-4’), 147.2 (C-4’’), 146.8 (C-7’, 7’’), 127.7 (C-1’, 1’’), 123.2 (C-6’, 6’’), 116.5 (C-5′, 5’’), 115.3 (C-8’), 115.0 (C-8’’), 114.9 (C-2’, 2’’), 78.3 (C-1), 74.0 (C-4), 70.0 (C-3), 67.1 (C-5), 37.9 (C-2), 35.7 (C-6)

3,5-Dicaffeoylquinic acid (**6**): Yellow amorphous powder; ESI-MS: 515.05 [M-H]^-^ (C_25_H_23_O_12_); ^1^H NMR (CD_3_OD, 400 MHz): δ 7.62 (1 H, d, *J* = 16.0 Hz, H-7’ or H-7’’), 7.58 (1 H, d, *J* = 16.0 Hz, H-7’ or H-7’’), 7.07 (2 H, br s, H-2’, H-2’’), 6.96 (2 H, m, H-6’, H-6’’), 6.78 (1 H, d, *J* = 8.0 Hz, H-5’, H-5’’), 6.35 (1 H, d, *J* = 16.0 Hz, H-8’ or H-8’’), 6.27 (1 H, d, *J* = 16.0 Hz, H-8’ or H-8’’), 5.43 (1 H, m, H-3), 5.39 (1 H, m, H-5), 3.97 (1 H, dd, *J* = 7.4, 3.1 Hz, H-4), 2.31–2.15 (4 H, m, H-2, H-6); ^13^ C NMR (CD_3_OD, 100 MHz): δ 177.7 (C-7), 168.9 (C-9’), 168.4 (C-9’’), 149.6 (C-4’), 149.5 (C-4’’), 147.3 (C-7’), 147.1 (C-7’’), 146.8 (C-3’, C-3’’), 127.9 (C-1’), 127.8 (C-1’’), 123.1 (C-6’), 123.0 (C-6’’), 116.5 (C-5’, C-5’’), 115.6 (C-2’’), 115.2 (C-2’), 115.1 (C-8’, C-8’’), 74.8 (C-1), 72.6 (C-5), 72.1 (C-3), 70.7 (C-4), 37.8 (C-2), 36.1 (C-6).

4,5-Dicaffeoylquinic acid (**7**): Yellow amorphous powder; ESI-MS: 515.05 [M-H]^-^ (C_25_H_23_O_12_); ^1^H NMR (DMSO-*d*_6_, 400 MHz): δ 7.48 (1 H, d, *J* = 15.9 Hz, H-7’), 7.42 (1 H, d, *J* = 15.9 Hz, H-7’’), 7.02 (2 H, br s, H-2’, H-2’’), 6.96 (2 H, s, H-6’, H-6’’), 6.74 (1 H, d, *J* = 8.0 Hz, H-5’, H-5’’), 6.23 (1 H, d, *J* = 16.0 Hz, H-8’), 6.15 (1 H, d, *J* = 16.0 Hz, H-8’’), 5.38 (1 H, m, H-5), 4.94 (1 H, br d, *J* = 6.6 Hz, H-4), 4.17 (1 H, m, H-3), 2.31–2.15 (4 H, m, H-2, H-6); ^13^C NMR (DMSO-*d*_6_, 100 MHz): δ 174.9 (C-7), 166.1 (C-9’’), 165.7 (C-9’), 148.5 (C-3’, C-3’’), 145.6 (C-4’, C-4’’), 145.5 (C-7’,C-7’’), 125.4 (C-1’, C-1’’), 121.5 (C-6’), 121.4 (C-6’’), 115.8 (C-2’), 115.7 (C-2’’), 114.8 (C-5’, C-5’’), 113.8 (C-8’), 113.6 (C-8’’), 73.7 (C-1), 71.5 (C-4), 67.7 (C-5), 66.7 (C-3), 37.5 (C-2), 35.9 (C-6).

3,4,5-Tricaffeoylquinic acid (**8**): Brown amorphous powder; ESI-MS: 677.16 [M-H]^-^ (C_34_H_29_O_15_); ^1^H NMR (400 MHz, CD_3_OD): δ 7.62 (1 H, d, *J* = 15.6 Hz, H-7’), 7.55 (1 H, d, *J* = 15.6 Hz, H-7’’), 7.53 (1 H, d, *J* = 15.6 Hz, H-7’’’), 7.05 (1 H, d, *J* = 1.8 Hz, H-2’), 7.01 (1 H, d, *J* = 1.8 Hz, H-2’’), 6.99 (1 H, d, *J* = 1.8 Hz, H-2’’’), 6.92 (1 H, dd, *J* = 8.4, 1.8 Hz, H-6’), 6.90 (1 H, dd, *J* = 8.4, 1.8 Hz, H-6’’), 6.85 (1 H, dd, *J* = 8.4, 1.8 Hz, H-6’’’), 6.78 (1 H, d, *J* = 8.4 Hz, H-5’), 6.76 (1 H, d, *J* = 8.4 Hz, H-5’’), 6.71 (1 H, d, *J* = 8.4 Hz, H-5’’’), 6.31 (1 H, d, *J* = 15.6 Hz, H-8’), 6.21 (1 H, d, *J* = 15.6 Hz, H-8’’), 6.19 (1 H, d, *J* =15.6 Hz, H-8’’’), 5.65 (2 H in total, m, H-3, 5), 5.32 (1 H, dd, *J* =8.4, 1.2 Hz, H-4), 2.45~2.20 (4 H in total, m, H-2, 6); ^13^C NMR (100 MHz, CD_3_OD): δ 174.2 (C-7), 168.5 (C-9’), 168.1 (C-9’’), 168.1 (C-9’’’), 149.7 (C-3’) 149.6 (C-3’’), 149.3 (C-3’’’), 147.9 (C-4’), 147.7 (C-4’’), 147.6 (C-4’’’), 146.7 (C-7’, 7’’, 7’’’), 127.8 (C-1’), 127.6 (C-1’’), 127.5 (C-1’’’), 123.3 (C-6’, 6’’, 6’’’), 116.5 (C-5’), 116.4 (C-5’’, 5’’’), 115.2 (C-8’), 115.1 (C-8’’), 115.1 (C-8’’’), 115.0 (C-2’), 114.6 (C-2’’), 114.3 (C-2’’’), 74.7 (C-1), 70.0 (C-4), 69.1 (C-3), 67.9 (C-5), 36.7 (C-2), 35.8 (C-6).

**Figure 3:**
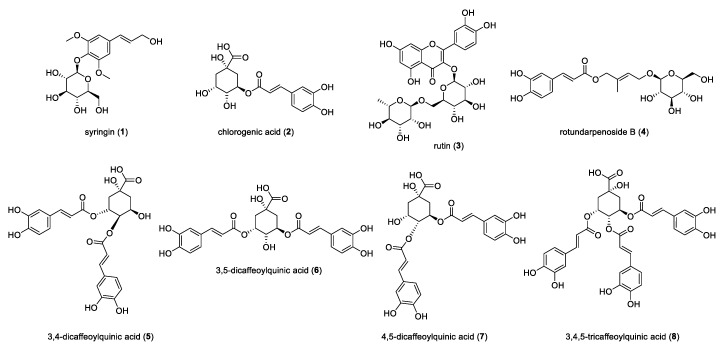
Structures of compounds **1**–**8** isolated from the extract of *I. rotunda* twigs.

Eight marker compounds were identified as syringin (**1**, *t*_R_ 14.19 min) [24], chlorogenic acid (**2**, *t*_R_ 15.69 min) [25], rutin (**3**, *t*_R_ 24.96 min) [26], rotundarepenoside B (**4**, *t*_R_ 27.73 min) [27], 3,4-dicaffeoylquinic acid (**5**, *t*_R_ 28.43 min) [28], 3,5-dicaffeoylquinic acid (**6**, *t*_R_ 30.96 min) [29], 4,5-dicaffeoylquinic acid (**7**, *t*_R_ 32.84 min) [30], and 3,4,5-tricaffeoylquinic acid (**8**, *t*_R_ 37.18 min) [31] based on combined spectroscopic analyses and comparison of spectroscopic data with those in the reference (Figure 3).

### 3.4. Method Validation of Marker Compounds (**1**–**8**) from I. rotunda

#### 3.4.1. Optimization of HPLC Condition

**Figure 4:**
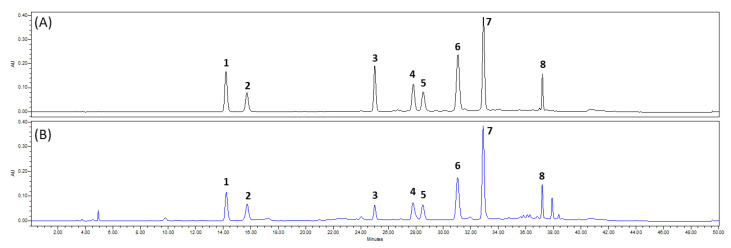
HPLC chromatograms of the eight marker compounds (**A**) and the extract of *I. rotunda* twigs (**B**) detected at 254 nm. Identified compounds are syringin (**1**; *t*_R_ 14.19 min), chlorogenic acid (**2**; *t*_R_ 15.69 min), rutin (**3**; *t*_R_ 24.96 min), rotundarpenoside B (**4**; *t*_R_ 27.73 min), 3,4-dicaffeoylquinic acid (**5**; *t*_R_ 28.43 min), 3,5-dicaffeoylquinic acid (**6**; *t*_R_ 30.96 min), 4,5-dicaffeoylquinic acid (**7**; *t*_R_ 32.84 min), and 3,4,5-tricaffeoylquinic acid (**8**; *t*_R_ 37.18 min).

The chromatographic profile of *I. rotunda* was obtained by optimizing analytical factors including column, mobile phase, gradient elution, flow rate, and wavelength detection. A Triart C_18_ column (4.6 × 250 mm, 5 μm) was chosen because it produced more selective and sharper peaks. A mobile phase with pure water containing 0.1% formic acid (A) and acetonitrile (B) was chosen and run according to the programmed gradient elution. Formic acid was the most effective buffer in the aqueous phase. This solvent system produced the high resolution of peak separation in the chromatograms. The column temperature was set at 35 °C to ensure precision. UV detection wavelengths were selected at 254 and 326 nm during experiments because these wavelengths were the most sensitive ones. Finally, the HPLC analytical method was successfully established. As shown in Figure 4, compounds **1**–**8** exhibited well-separated peaks with a high resolution. Thus, this optimal chromatographic condition was employed to validate marker compounds **1**–**8** obtained from the extract of *I. rotunda* twigs.

#### 3.4.2. Method Validation of Quantitative Analysis

HPLC experiments for linearity, precision, and repeatability were performed to ensure that the present method was sensitive, selective, precise, and accurate. Subsequently, the established method was used to quantify the eight marker compounds obtained from the extract of *I. rotunda* twigs.

##### Linearity, LODs, and LOQs

The linearity was measured based on values of correlation coefficients (R^2^) using calibration curves of each compound. The linearity of the eight compounds showed the best R^2^ values (≥ 0.9993) with the following concentration ranges: 6.25–200 μg/mL for **1** and **3**; 12.5–400 μg/mL for **2**, **4**, **5** and **8**; and 25–800 μg/mL for **6** and **7**. The LOD and LOQ of these eight compounds were 0.13–0.65 and 0.42–1.98 μg/mL, respectively (Table 1).

##### Precision, Accuracy, and Recovery

To evaluate the recovery, three different amounts (low, medium, and high) were spiked to the *I. rotunda* sample. Accuracy was assessed by measuring the mean recovery (%) of standard compounds from the spiked extract solution versus the nonspiked extract sample. As a results, recoveries of these eight compounds were in the range of 96.60–104.7% (Table 2), demonstrating that the developed method was suitable for assessing these marker compounds in *I. rotunda*. The repeatability was performed by analyzing eight independently prepared samples using the same method. To evaluate the precision of this method, we determined intra- and interday RSD values. RSD values of intraday and interday evaluations (n = 6) were 0.40–1.15 and 2.48–3.65%, respectively (Table 2).

##### Quantification of Marker Compounds in *I. rotunda*

The above-established HPLC validation method was used to quantitate content of marker compounds in the crude extract of *I. rotunda* twigs. Compound **7** (4,5-dicaffoylquinic acid) showed the highest content (93.43 mg/g) in the twig extract, followed by compound **6** (3,5-dicaffoylquinic acid) at 72.77 mg/g, compound **8** (3,4,5-tricaffoylquinic acid) at 50.02 mg/g, compound **2** (chlorogenic acid) at 38.54 mg/g, compound **5** (3,4-dicaffoylquinic acid) at 35.45 mg/g, compound **4** (rotundarpenoside B) at 35.43 mg/g, compound **1** (syringin) at 16.72 mg/g, and compound **3** (rutin) at 8.62 mg/g, respectively (Table S1).

### 3.5. Screening of Antioxidants by HPLC-DPPH Method and ELISA Assay 

**Figure 5:**
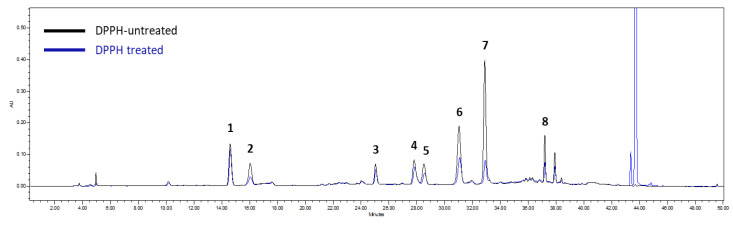
Chromatogram of HPLC-DPPH for screening antioxidants from the extract of *I. rotunda* twigs. Compounds **1**–**8** were identified as antioxidants by the HPLC-DPPH screening method. The HPLC peak areas of these eight marker compounds reduced after reaction with DPPH radicals (DPPH group) compared with those from the DPPH-free group.

The HPLC-DPPH method can be used to rapidly assess pure antioxidant compounds in complex mixtures [17]. The above-established method was used to determine antioxidant compounds based on reduced peak areas between DPPH treated and untreated groups. As shown in Figure 5, compound **7** (4,5-dicaffoylquinic acid) showed the highest antioxidant capacity with a reduction peak area of 83.67%. Compound **8** (3,4,5-tricaffoylquinic acid), compound **2** (chlorogenic acid), and compound **6** (3,5-dicaffoylquinic acid) showed significant antioxidant activities with reduction peak areas of 67.25%, 60.51%, and 58.88%, respectively (Figure 5).

Subsequently, antioxidant activity of the most active marker compounds (**2** and **6**–**8**) was further verified using ELISA. Results revealed that compounds **6**–**8** exhibited significant antioxidant activities with EC_50_ values ranging from 10.88 to 13.84 μM, stronger than compound **2** with EC_50_ value of 35.50 μM. (Table 3). 

### 3.6. Screening of Brain-Penetrable Antioxidants by PAMPA-BBB Method

**Figure 6:**
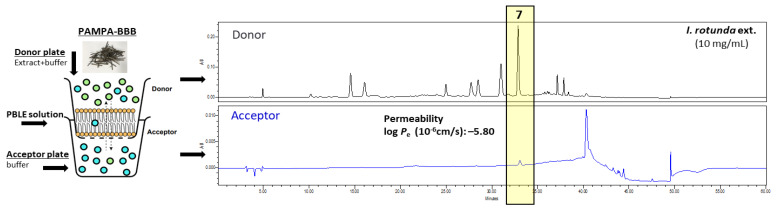
Result of the PAMPA-BBB experiment of the extract of *I. rotunda* twigs. Compound **7** detected in donor and acceptor wells.

Permeability assessment of small molecules through the blood–brain barrier (BBB) plays a significant role in the development of effective central nervous system (CNS) drug candidates [32]. For this purpose, to investigate brain-penetrable antioxidants from *I. rotunda* extract, PAMPA-BBB assay was conducted. Coupling PAMPA-BBB to the above-established HPLC method allowed rapid and simultaneous investigation of membrane penetration capabilities of compounds present in the *I. rotunda* extract. As seen in Figure 6 and Figure S19, the 4,5-dicaffoylquinic acid (**7**) was detected in the acceptor solution with BBB permeability log *P*_e_ value of −5.80, showing PAMPA-BBB potential penetrability based on the study of Könczöl et al. [22]. These findings were in good agreement with the log *P*_e_ range for classifying CNS drug candidates with moderate BBB permeation potential (- = not detected in acceptor or log *P*_e_ < −6.0; + = log *P*_e_ > −6.0; ++ = log *P*_e_ > −5.0) [22].

Subsequently, compounds **1**–**8** were further tested using the PAMPA-BBB method at the same concentration of 10 mM because the content of each compound was not consistent in the extract solution. Coumarin and caffeic acid were positive and negative controls, respectively. As can be seen in Table 4 and Figure S20, compound **7** (4,5-dicaffoylquinic acid) showed similar log *P*_e_ value of −5.85 compared to previous experiment, whereas compounds **1**–**6** and **8** were not detected in the acceptor solution. Coumarin and caffeic acid showed log *P*_e_ values of −4.54 and −9.08, respectively (Table 4). Thus, compound **7** was finally demonstrated to have a moderate BBB permeability.

## 4. Discussion

*Ilex rotunda* Thunb., the herbal medicine “Jiubiying”, is widely used as a traditional Chinese medicine for reducing fever, relieving pain, indigestion, and analgesia [33]. A previous study has isolated large amounts of triterpenes and triterpene glycosides from *I. rotunda* fruits and leaves [14]. Although some *Ilex* species have been reported as sources of antioxidants, information about antioxidative phytochemicals from *I. rotunda* is still limited. Thus, we tried to discover potential antioxidant agents from *I. rotunda* extract and further evaluate their blood–brain barrier permeability using the PAMPA-BBB method.

At first, radical scavenging effects of the extracts of *I. rotunda* fruits, twigs, and leaves were evaluated using DPPH and ABTS assays. Results revealed that the twig extract (45.9%) exhibited stronger radical scavenging activity than the fruit extract (42.4%) and leaf extract (42.2%) at 50 µg/mL (Figure 1) using DPPH assay. In the ABTS assay, the twig extract and leaf extract showed similar radical scavenging activities. Finally, we selected its twig extract in consideration of yield of compounds. Next, we optimized the extraction condition using 80% EtOH based on DPPH and ABTS assays (Figure S2). The 80% EtOH extract of twig was then successfully partitioned into *n*-hexane, chloroform, EtOAc, *n*-BuOH, and aqueous fractions. To identify fractions with strong activities, free radical scavenging activities of fractions were evaluated. It was found that the *n*-BuOH fraction showed the most potent antioxidant activity (Figure 2). Thus, chemical constituents of this fraction was extensively investigated, leading to the isolation of eight marker compounds (**1**–**8**) (Figure 3). 

Subsequently, an analytical method was established based on the eight marker compounds (**1**–**8**). This analytical method was adapted to optimize analytical factors with a high resolution and efficiency (Figure 4). It was then applied to simultaneous determination of eight marker compounds: syringin (**1**), chlorogenic acid (**2**), rutin (**3**), rotundarpenoside B (**4**), 3,4-dicaffeoylquinic acid (**5**), 3,5-dicaffeoylquinic acid (**6**), 4,5-dicaffeoylquinic acid (**7**), and 3,4,5-tricaffeoylquinic acid (**8**) at amounts of 8.62–93.43 mg/g. The established method was validated to have appropriate sensitivity, repeatability, and precision (Table 1, Table 2, and Table S1).

The above analytical method was also employed to screen antioxidant properties of marker compounds derived from twig extract via peak areas reduction using a screening HPLC-DPPH method. Natural antioxidants often decrease during their isolation and purification due to decomposition [34]. An HPLC-DPPH method combining separation and activity evaluation would present a major advantage for rapid screening antioxidant constituents of extract solution. In this experiment, compound **7** showed a higher (83.67%) peak area reduction than those with DPPH-free group (Figure 5). In contrast, compounds **8**, **2**, and **6** exhibited strong DPPH radical scavenging activities with peak area reduction ranging from 58.88% to 67.25% (Table 3). These active compounds were further verified using ELISA. As shown in Table 3, compounds **6**–**8** exhibited significant antioxidant activity with EC_50_ value ranging from 10.88 to 13.84 μM, stronger than compound **2** with EC_50_ value of 35.50 μM. These results suggested that the highest peak area reduction of **7** in the HPLC-DPPH method was influenced by the highest content value (93.43 mg/g for **7**) in *I. rotunda* extract. The HPLC-DPPH method can provide bioactive evaluation and quantitative information [35]. 

Antioxidant effects of active compounds **2** and **6**–**8** can also be found in the following literature. Chlorogenic acid (CGA) (**2**) is widely recognized to have antioxidant activity. It exists in most abundant quantity in different foods, coffee, and vegetables [36]. An intake of CGAs through coffee drinking has many beneficial effects on human health, such as antioxidative, anticarcinogenic, and antibacterial effects [37]. 3,4-Dihydroxyl group of CGA might donate hydrogen atoms for following oxidation to respective phenoxyl radicals. These radicals are quickly stabilized by resonance stabilization. As a result, this reaction reduced free radicals and inhibited oxidation reactions [38]. Three isomeric compounds of CGA (3,5-di-CQA (**6**), 4,5-di-CQA (**7**), and 3,4,5-tri-CQA (**8**)) also reported as antioxidant due to the presence of high numbers of 3,4-dihydroxyl groups [39]. As shown in Table 3, compounds **6**–**8** showed stronger antioxidant activities than compound **2**, suggesting that the presence of more 3,4-dihydroxy moiety contributed to the free radicals scavenging ability.

Furthermore, parallel artificial membrane permeability assay for the blood–brain barrier (PAMPA-BBB) was applied to investigate brain-penetrable antioxidants from *I. rotunda* extract. The brain with a high oxygen consumption is highly sensitive to oxidative stress [12]. When ROS production rises over the limit of the scavenging capacity of the antioxidant response system, extensive protein oxidation and lipid peroxidation will occur, causing oxidative damage [40]. Natural products possess a high chemical scaffold diversity. They have been historically proven to be rich sources of various antioxidants. However, most compounds showed a poor blood–brain barrier (BBB) permeability [41]. For this purpose, the PAMPA-BBB assay was chosen to further investigate brain-penetrable antioxidants from the *I. rotunda* extract. As shown in Figure 6, compound **7** (4,5-dicaffoylquinic acid) was detected in the acceptor solution. Subsequently, the log *P*_e_ value for **7** was calculated by the above-described equation. The concentrations of acceptor (*C*_A(*t*)_ = 6.62 μg/mL) and donor (*C*_D(*t*)_ = 287.86 μg/mL) solutions were calculated based on the peak area and regression equation for **7**, respectively. Other parameters were used as follows: *A* = 0.3 cm^2^, *V*_D_ = 150 μL, *V*_A_ = 300 μL, and *t* =14,400 s. Finally, the log *P*_e_ value (−5.80) for **7** was determined. Compounds **1**–**6** and **8** were not detected in the acceptor area. Thus, the permeability values were not calculated. The detailed calculation procedure can also be found in the supplementary data (Figure S19). In the same concentration (10 mM) test, compound **7** also showed similar log *P*_e_ value of −5.85, whereas other compounds (**1**–**6**, and **8**) were not detected (Table 4). Thus, compound **7** was confirmed to have a moderate BBB permeability. A previous study showed that chlorogenic acid (**2**) and rutin (**4**) have poor permeability [21]. Other compounds (**1**, **3**, and **5**–**8**) were firstly tested for BBB permeability in this experiment. Some authors have noted the importance of polar surface area (PSA), lipophilicity, molecular weight, and hydrogen bond donors in natural molecules for BBB permeability [42]. Nevertheless, other molecular factors can also affect BBB diffusion, such as Hansen polarity, topological polar surface area (TPSA), and optimal (PK) properties [43]. For these reasons, 4,5-dicaffoylquinic acid (**7**) with a high molecular weight can also cross the BBB, although less efficiently. 

## 5. Conclusions

In conclusion, we evaluated antioxidant effects of fractions and compounds from the extract of *I. rotunda* twigs by measuring DPPH and ABTS radical scavenging assays. BuOH fraction showed the most potent inhibitory activity. It subsequently afforded eight marker compounds (**1**–**8**) via isolation and structure determination. The established method was successfully applied to quantify levels of marker compounds and applied to evaluate their antioxidant activities with a rapid screening HPLC-DPPH method. Significant active marker compounds **2** and **6**–**8** were further verified using ELISA. Furthermore, the PAMPA-BBB method was applied to investigate brain-penetrable antioxidants from the *I. rotunda* extract. As a result, 4,5-dicaffeoylquinic acid (**7**) was able to penetrate across the blood–brain barrier via transcellular passive diffusion. Our findings suggest that compound **7** can be used as a therapeutic potential candidate in natural product-based CNS (central nervous system) drug discovery. Further in silico modeling and in vivo study are needed in the future to better understand the exact mechanisms of action of this compound

## Figures and Tables

**Table 1:** Concentration ranges, regression equation, LODs, and LOQs, of the eight marker components in the extract of *I. rotunda* twigs.

Marker Compound	Concentration Range (µg/mL)	*^a^* Regression Equation	*^b^* Correlation Coefficient (R^2^)	*^c^* LOD (µg/mL)	*^d^* LOQ (µg/mL)
syringin (**1**)	6.25 ~ 200	y = 11,125x + 16,180	0.9999	0.18	0.55
chlorogenic acid (**2**)	12.5 ~ 400	y = 2977.6x + 10,941	0.9993	0.45	1.38
rutin (**3**)	6.25 ~ 200	y = 10,785x + 18,732	0.9998	0.13	0.42
rotundarpenoside B (**4**)	12.5 ~ 400	y = 4358.5x + 4988.8	0.9999	0.26	0.81
3,4-dicaffeoylquinic acid (**5**)	12.5 ~ 400	y = 3462.4x − 8499.8	0.9995	0.65	1.98
3,5-dicaffeoylquinic acid (**6**)	25 ~ 800	y = 4918.7x − 13,540	0.9998	0.38	1.18
4,5-dicaffeoylquinic acid (**7**)	25 ~ 800	y = 5893x − 25,506	0.9993	0.27	0.78
3,4,5-tricaffeoylquinic acid (**8**)	12.5 ~ 400	y = 2617.1x + 916.7	0.9999	0.58	1.76

*^a^* y: peak area at 254 and 326 nm; x: concentration (μg/mL) of compounds; *^b^* R^2^, correlation coefficient for 6 data points in the calibration curves (*n* = 3); *^c^* LOD: 3.3 × SD/S; *^d^* LOQ: 10 × SD/S. SD is the standard deviation.

**Table 2:** Recovery data for the eight marker components in the extract of *I. rotunda* twigs.

Marker Compound	Concentration Range (µg/mL)	*^a^* Recovery (%)	*^b^* Precision (RSD %)	
			Intraday	Interday
syringin (**1**)	40	96.60	0.40	2.48
	100	97.60		
	200	95.30		
chlorogenic acid (**2**)	64	104.62	1.15	3.65
	160	102.73		
	400	104.53		
rutin (**3**)	40	104.03	0.46	2.53
	100	102.10		
	200	100.47		
rotundarpenoside B (**4**)	64	103.92	0.85	3.27
	160	99.78		
	400	102.58		
3,4-dicaffeoylquinic acid (**5**)	64	104.7	0.51	2.81
	160	97.26		
	400	100.21		
3,5-dicaffeoylquinic acid (**6**)	128	99.93	0.47	2.70
	320	98.18		
	800	99.09		
4,5-dicaffeoylquinic acid (**7**)	128	99.44	0.68	3.04
	320	97.88		
	800	98.79		
3,4,5-tricaffeoylquinic acid (**8**)	64	100.89	0.47	2.74
	160	100.32		
	400	103.6		

*^a^* Recovery (%) = (detected concentration × 100/(original concentration + spiked concentration), *^b^* Precision is expressed as RSD (%) = (SD/mean) × 100.

**Table 3:** Antioxidant effect of eight marker compounds on DPPH radical.

Marker Compounds	*^a^* Reduction of the Peak Area (%)	EC_50_ Values (μM)
syringin (**1**)	6.98 ± 0.44	–
chlorogenic acid (**2**)	60.51 ± 0.31	35.50 ± 0.38
rutin (**3**)	21.45 ± 0.90	–
rotundarpenoside B (**4**)	18.99 ± 0.90	–
3,4-dicaffeoylquinic acid (**5**)	45.24 ± 0.67	–
3,5-dicaffeoylquinic acid (**6**)	58.88 ± 0.44	10.88 ± 0.04
4,5-dicaffeoylquinic acid (**7**)	83.67 ± 0.19	13.84 ± 0.24
3,4,5-tricaffeoylquinic acid (**8**)	67.25 ± 1.00	10.89 ± 0.14
Ascorbic acid *	–	22.56 ± 0.77

* Positive control. *^a^* Reduction of peak areas between DPPH treated and untreated samples in the *I. rotunda* extract. Peaks area of the untreated DPPH sample was considered as 100%.

**Table 4:** Results for BBB permeability of eight marker compounds (**1**–**8**) at concentration of 10 mM.

Marker Compounds	BBB Permeability log *P*_e_ (cm/s)	Cross BBB Potential *^a^*
syringin (**1**)	*^b^* n.d.	-
chlorogenic acid (**2**)	n.d.	-
rutin (**3**)	n.d.	-
rotundarpenoside B (**4**)	n.d.	-
3,4-dicaffeoylquinic acid (**5**)	n.d.	-
3,5-dicaffeoylquinic acid (**6**)	n.d.	-
4,5-dicaffeoylquinic acid (**7**)	−5.85 ± 0.01	+
3,4,5-tricaffeoylquinic acid (**8**)	n.d.	-
*^c^* coumarin	−4.54 ± 0.01	++
*^d^* caffeic acid	−9.08 ± 0.01	-

*^a^* PAMPA-BBB potential penetrability based on Könczöl et al.; − = not detected in acceptor or log *P*_e_ < −6.0; + = log *P*_e_ > −6.0; ++ = log *P*_e_ > −5.0 [22]. *^b^* n.d. = not detected. *^c^* Positive control. *^d^* Negative control.

## Data Availability

The data presented in this study are available in the article or Supplementary Materials.

## Supplementary Materials

The following supporting information can be downloaded at: https://www.mdpi.com/article/10.3390/antiox11101989/s1, Figure S1: UV and mass spectra of marker compounds (**1**–**8**); Figure S2: DPPH (A) and ABTS (B) radical scavenging effects of the extract of *I. rotunda* twigs on various solvent ratios; Table S1: Contents of eight marker compounds (**1**–**8**) in the extract of *I. rotunda* twigs; Figure S3–S18: ^1^H and ^13^C NMR spectra of compounds **1**–**8**; Figure S19: PAMPA-BBB permeability test result for *I. rotunda* ext. (10 mg/mL) and detailed calculation procedure of permeability value for compound **7**; Figure S20: PAMPA-BBB permeability test results for compounds **1**–**8** (10 mM).
