# Supplementary material for: Analysis of Antioxidant Constituents from Ilex rotunda and Evaluation of Their Blood–Brain Barrier Permeability
Source: Antioxidants (Basel). 2022 Oct 6;11(10):1989. doi: 10.3390/antiox11101989 (PMC9598469; doi:10.3390/antiox11101989)
Supplement: Supplementary file 1 [file antioxidants-11-01989-s001.zip › antioxidants-1933896-supplementary.pdf]

Supplementary data

Analysis of Antioxidant Constituents from *Ilex rotunda* and Evaluation of Their Blood-Brain Barrier Permeability

Chang-Kwon Kim, Jeongjun Ahn, Jayeon Yu, DucDat Le, Sanghee Han, and Mina Lee*

College of Pharmacy, Sunchon National University, 255 Jungangno, Suncheon-si 57922, Jeonnam, Republic of Korea

Contents

Figure S1. UV and mass spectra of marker compounds (**1-8**) S3
Figure S2. DPPH (A) and ABTS (B) radical scavenging effects of the extract of *I. rotunda* twigs on various solvent ratios S4
Table S1. Contents of eight marker compounds (**1-8**) in the extract of *I. rotunda* twigs S5
Figure S3. ^{1}H NMR (400 MHz, MeOH-d_4) spectrum of **1** S6
Figure S4. ^{13}C NMR (100 MHz, MeOH-d_4) spectrum of **1** S6
Figure S5. ^{1}H NMR (400 MHz, MeOH-d_4) spectrum of **2** S7
Figure S6. ^{13}C NMR (100 MHz, MeOH-d_4) spectrum of **2** S7
Figure S7. ^{1}H NMR (400 MHz, MeOH-d_4) spectrum of **3** S8
Figure S8. ^{13}C NMR (100 MHz, MeOH-d_4) spectrum of **3** S8
Figure S9. ^{1}H NMR (400 MHz, MeOH-d_4) spectrum of **4** S9
Figure S10. ^{13}C NMR (100 MHz, MeOH-d_4) spectrum of **4** S9
Figure S11. ^{1}H NMR (400 MHz, MeOH-d_4) spectrum of **5** S10
Figure S12. ^{13}C NMR (100 MHz, MeOH-d_4) spectrum of **5** S10
Figure S13. ^{1}H NMR (400 MHz, MeOH-d_4) spectrum of **6** S11
Figure S14. ^{13}C NMR (100 MHz, MeOH-d_4) spectrum of **6** S11
Figure S15. ^{1}H NMR (400 MHz, MeOH-d_4) spectrum of **7** S12
Figure S16. ^{13}C NMR (100 MHz, MeOH-d_4) spectrum of **7** S12
Figure S17. ^{1}H NMR (400 MHz, MeOH-d_4) spectrum of **8** S13
Figure S18. ^{13}C NMR (100 MHz, MeOH-d_4) spectrum of **8** S13
Figure S19. PAMPA-BBB permeability test result for *I. rotunda* ext. (10 mg/ml) and detailed calculation procedure of permeability value for compound **7** S14
Figure S20. PAMPA-BBB permeability test results for compounds **1-8** (10 mM) S15

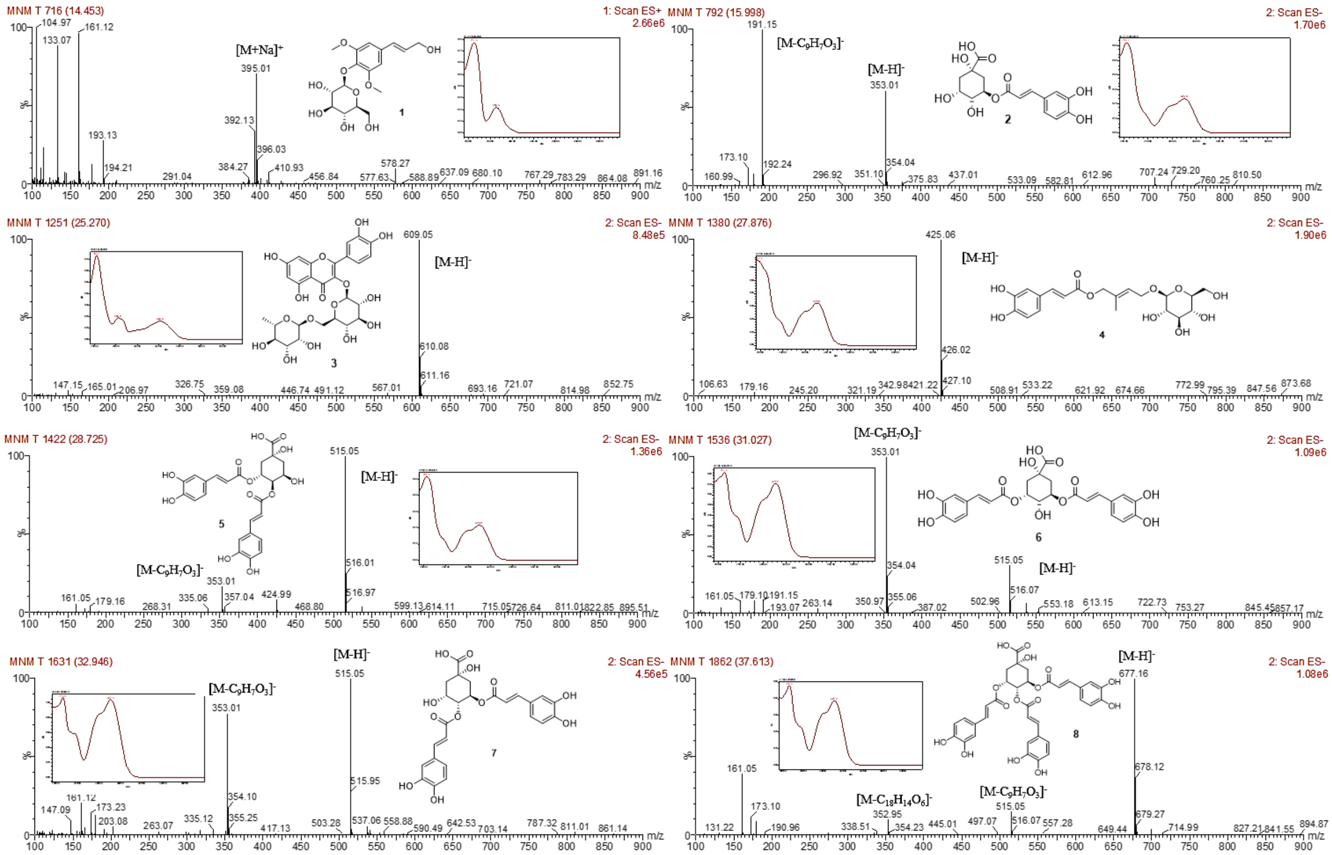

Figure S1. UV and mass spectra of marker compounds (**1**-**8**)

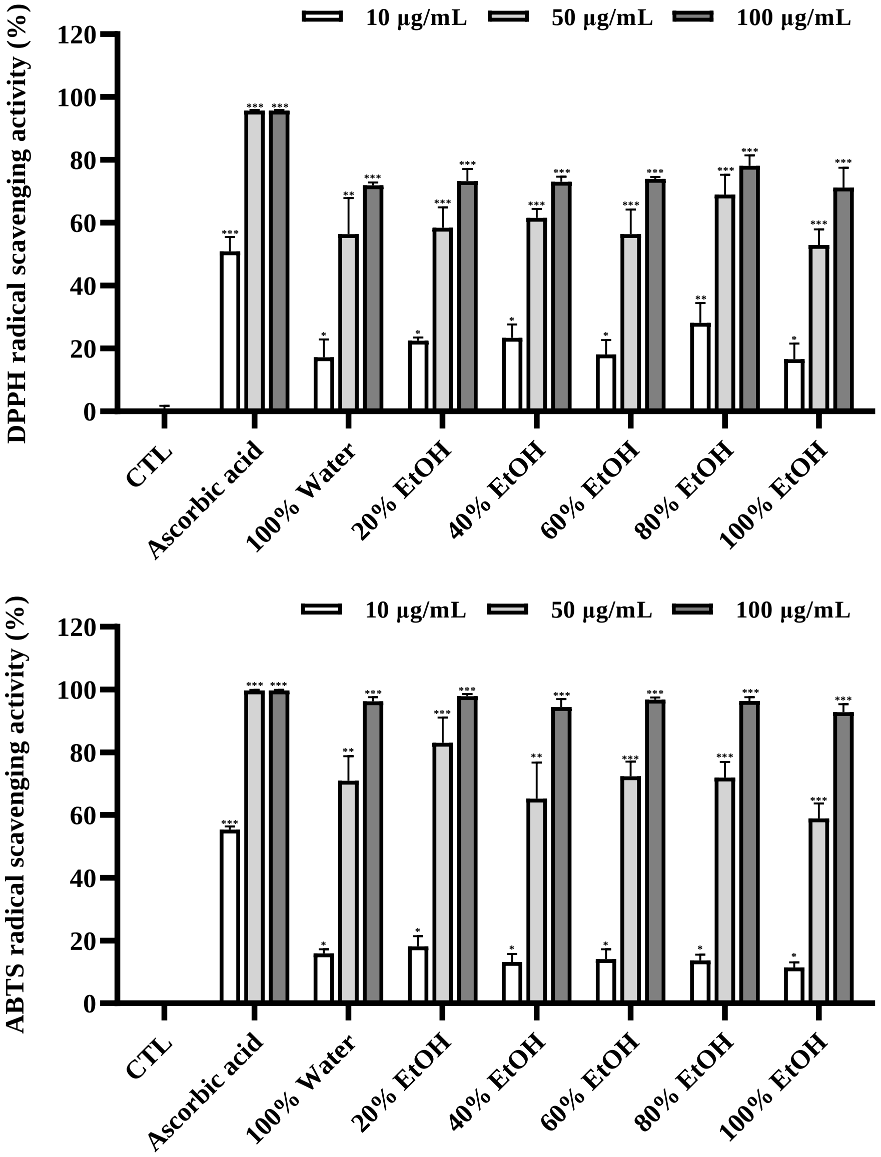

Figure S2. DPPH (A) and ABTS (B) radical scavenging effects of the extract of *I. rotunda* twigs on various solvent ratios. The data are expressed as the mean ± SD (n = 3) of three individual experiments. * $p < 0.05$, ** $p < 0.01$ and *** $p < 0.001$, compared with control.

Table S1. Contents of eight marker compounds (**1-8**) in the extract of *I. rotunda* twigs.

**Marker compounds**	**Amount (ppm)**	**Content (mg/g)**
syringin (**1**)	112.04 ± 0.45	16.72
chlorogenic acid (**2**)	258.25 ± 2.71	38.54
rutin (**3**)	57.74 ± 0.24	8.62
rotundarpenoside B (**4**)	237.41 ± 1.83	35.43
3,4-dicaffeoylquinic acid (**5**)	237.58 ± 1.10	35.45
3,5-dicaffeoylquinic acid (**6**)	487.67 ± 2.13	72.77
4,5-dicaffeoylquinic acid (**7**)	626.10 ± 3.88	93.43
3,4,5-tricaffeoylquinic acid (**8**)	335.2 ± 1.43	50.02

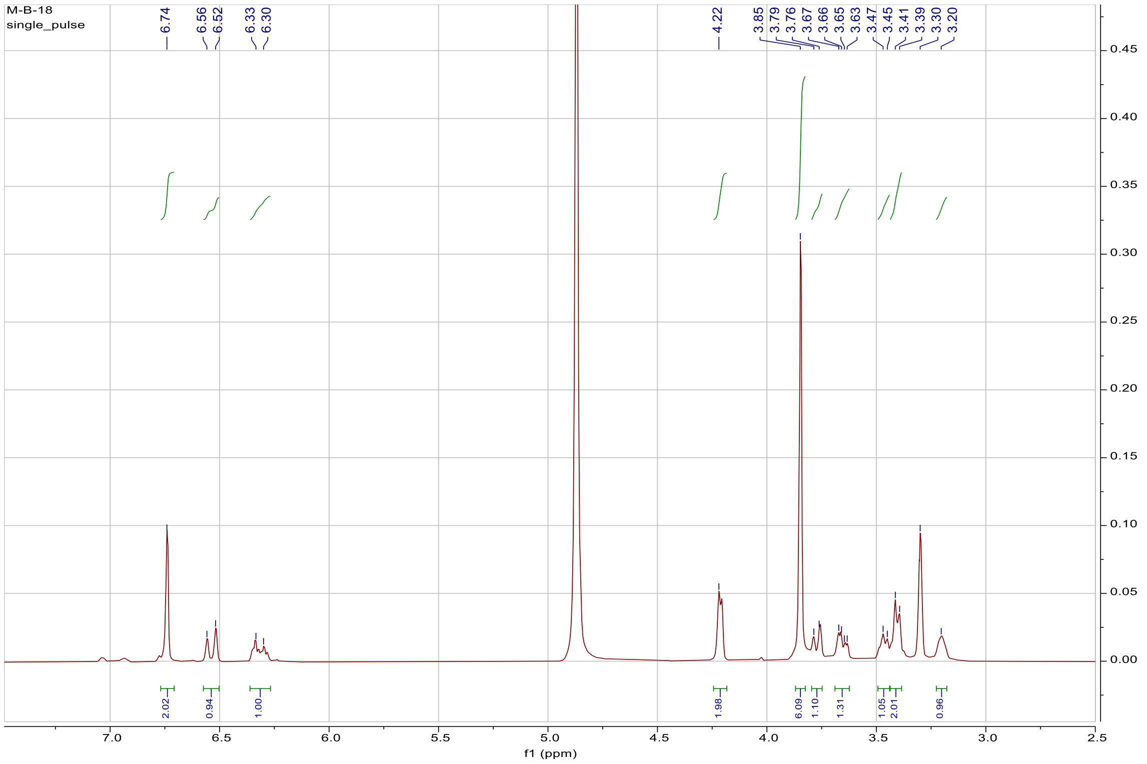

Figure S3. ^{1}H NMR (400 MHz, MeOH-d_4) spectrum of **1**

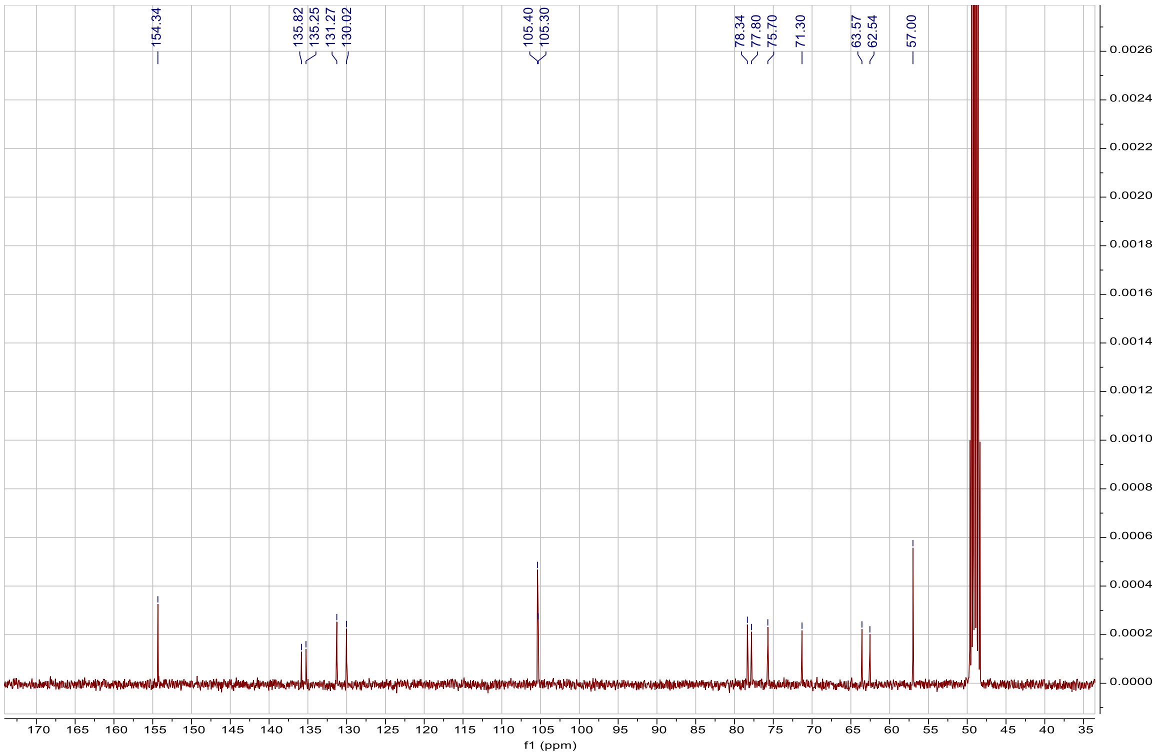

Figure S4. ^{13}C NMR (100 MHz, MeOH-d_4) spectrum of **1**

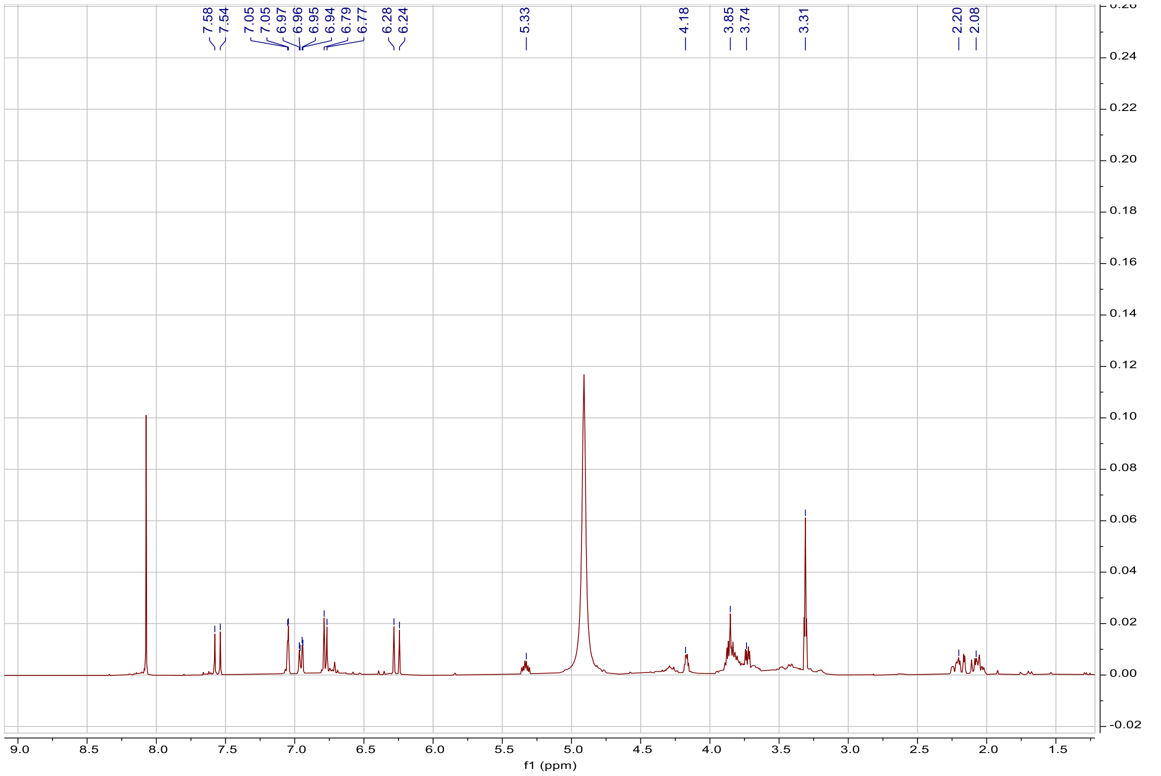

Figure S5. ^{1}H NMR (400 MHz, MeOH-d_4) spectrum of **2**

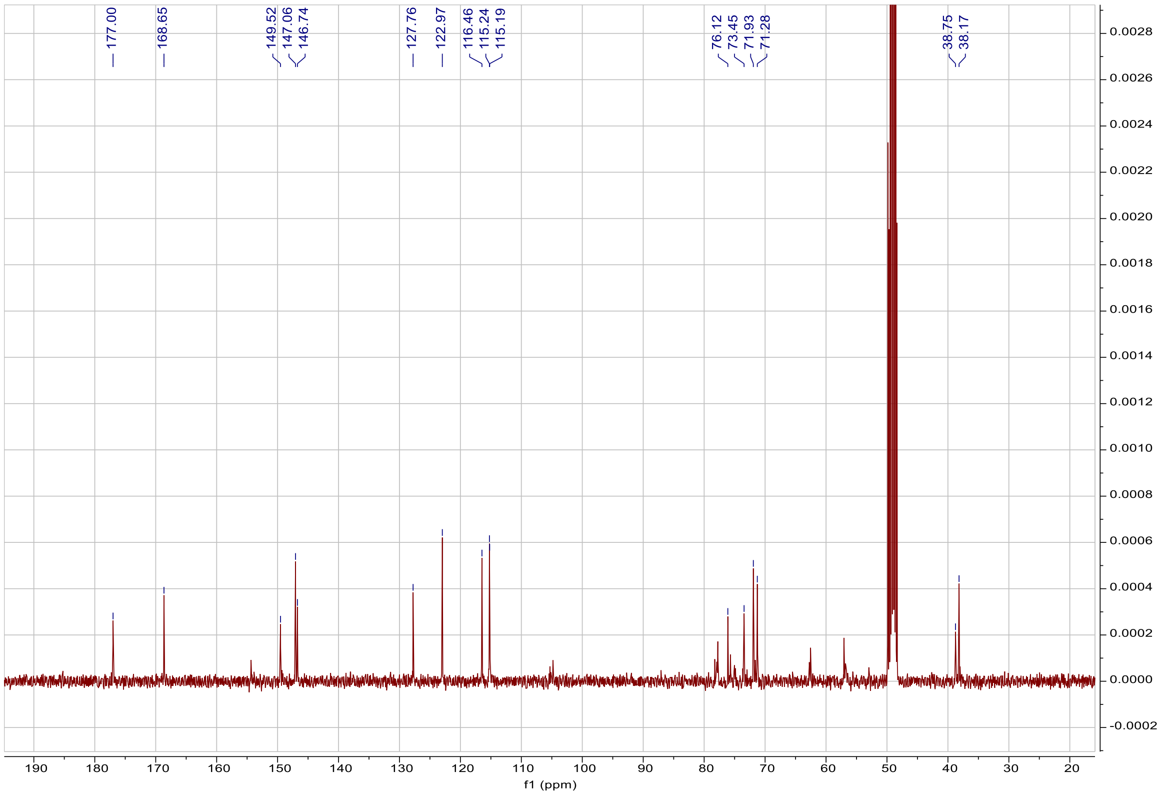

Figure S6. ^{13}C NMR (100 MHz, MeOH-d_4) spectrum of **2**

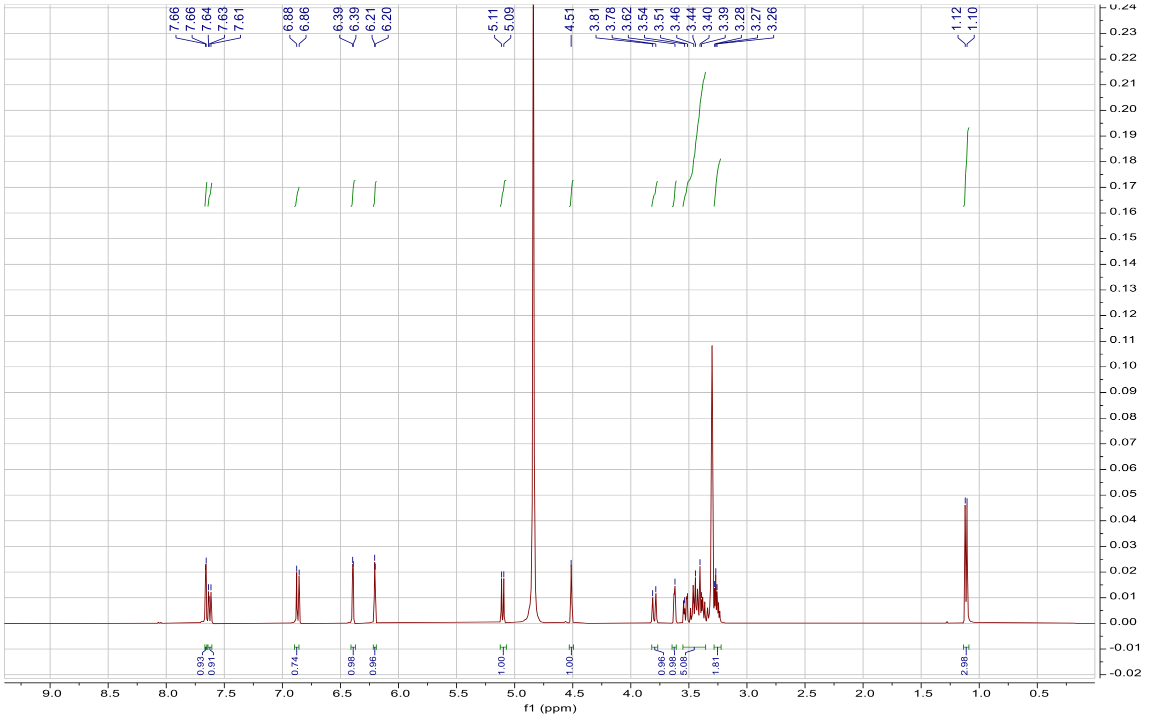

Figure S7. ^{1}H NMR (400 MHz, MeOH-d_4) spectrum of **3**

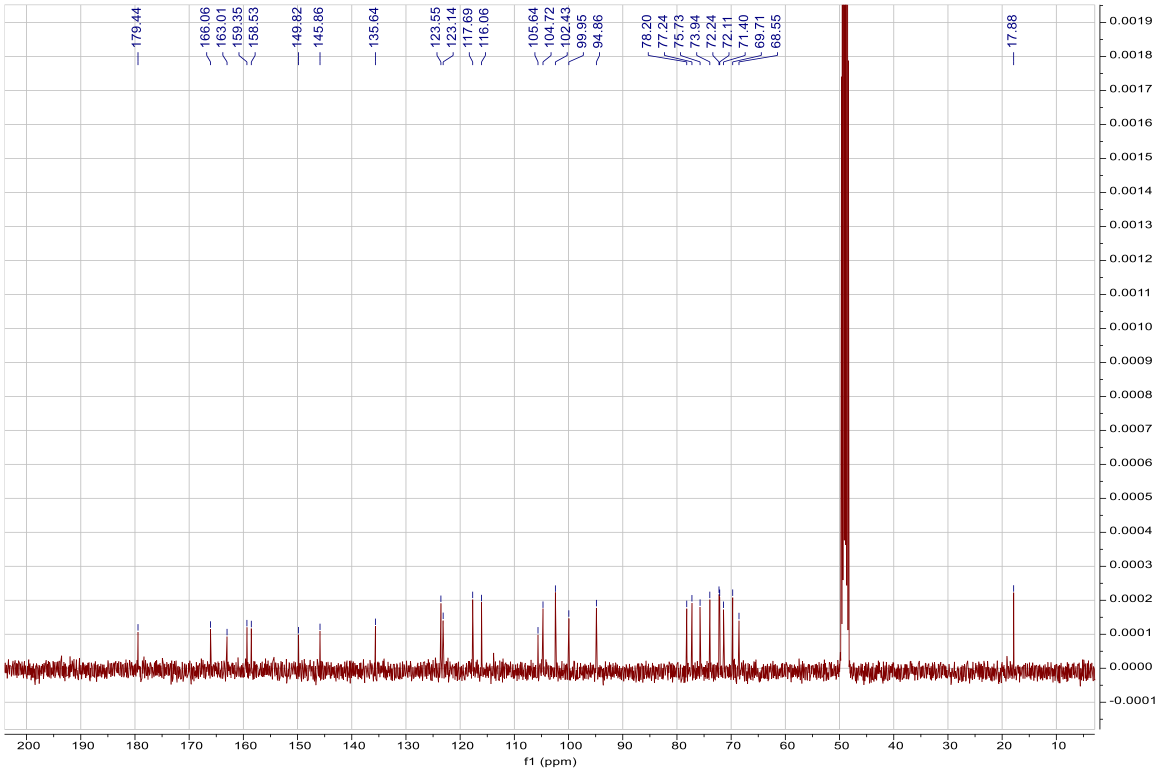

Figure S8. ^{13}C NMR (100 MHz, MeOH-d_4) spectrum of **3**

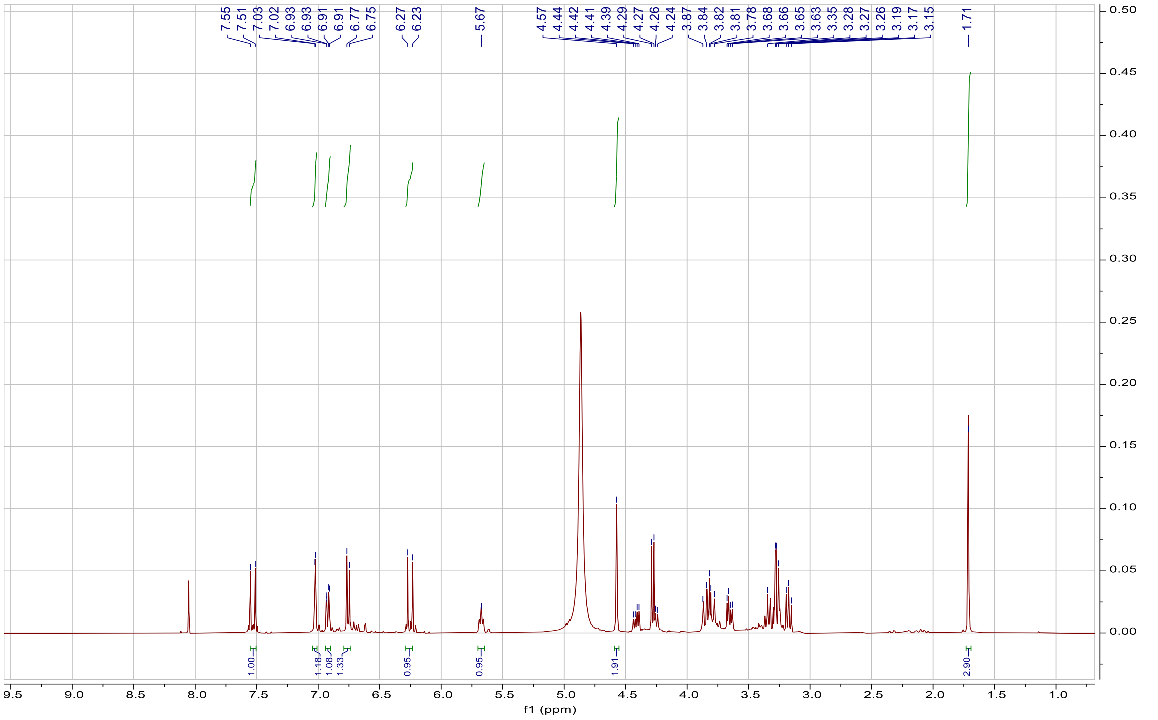

Figure S9. ^{1}H NMR (400 MHz, MeOH-d_4) spectrum of **4**

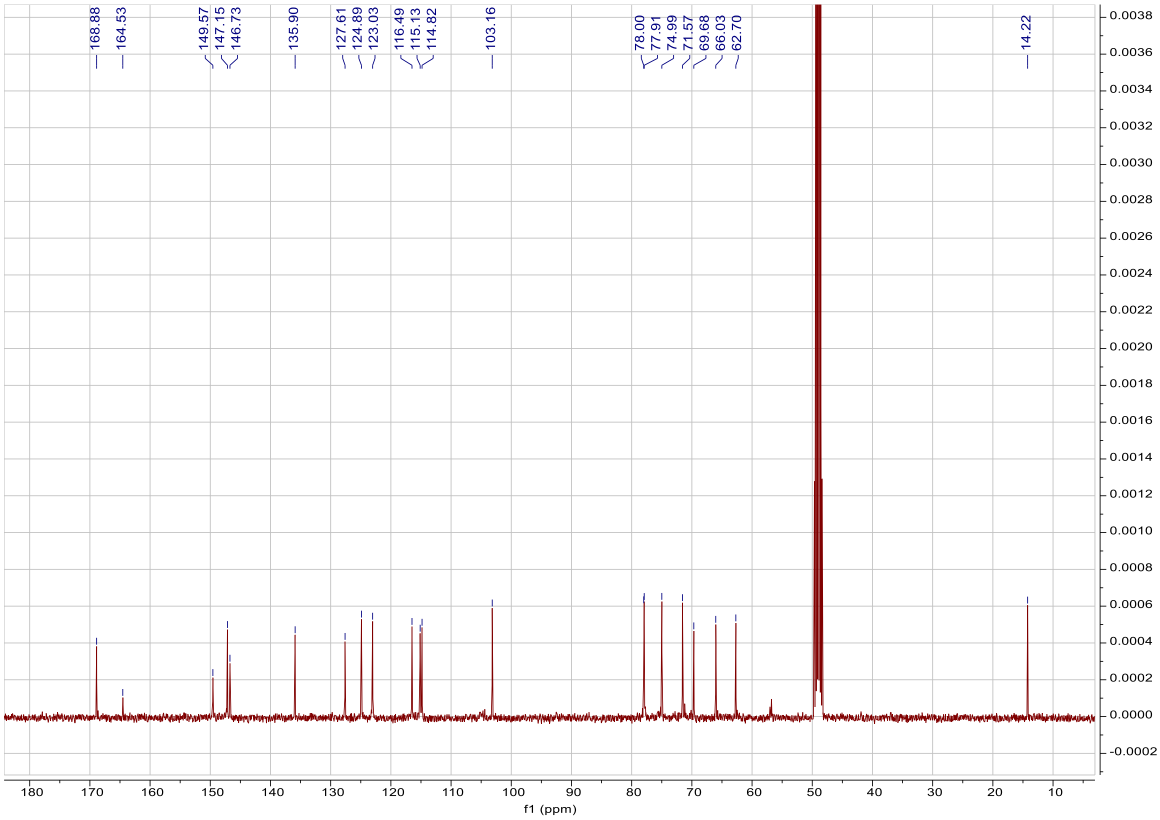

Figure S10. ^{13}C NMR (100 MHz, MeOH-d_4) spectrum of **4**

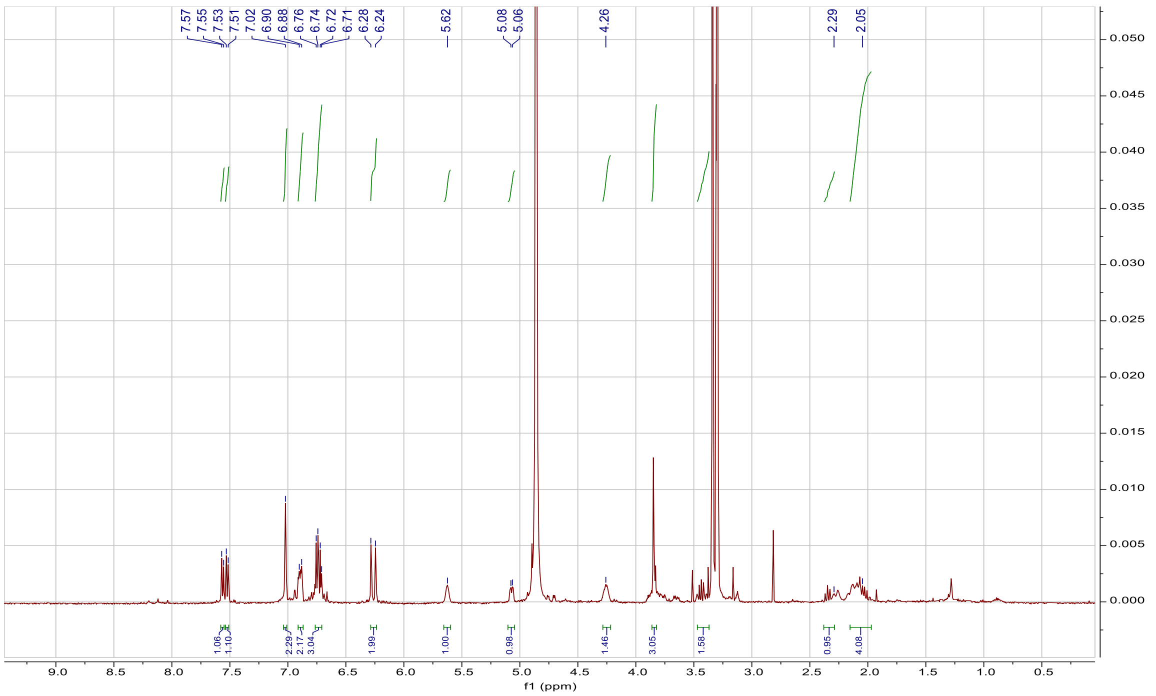

Figure S11. ^{1}H NMR (400 MHz, MeOH-d_4) spectrum of **5**

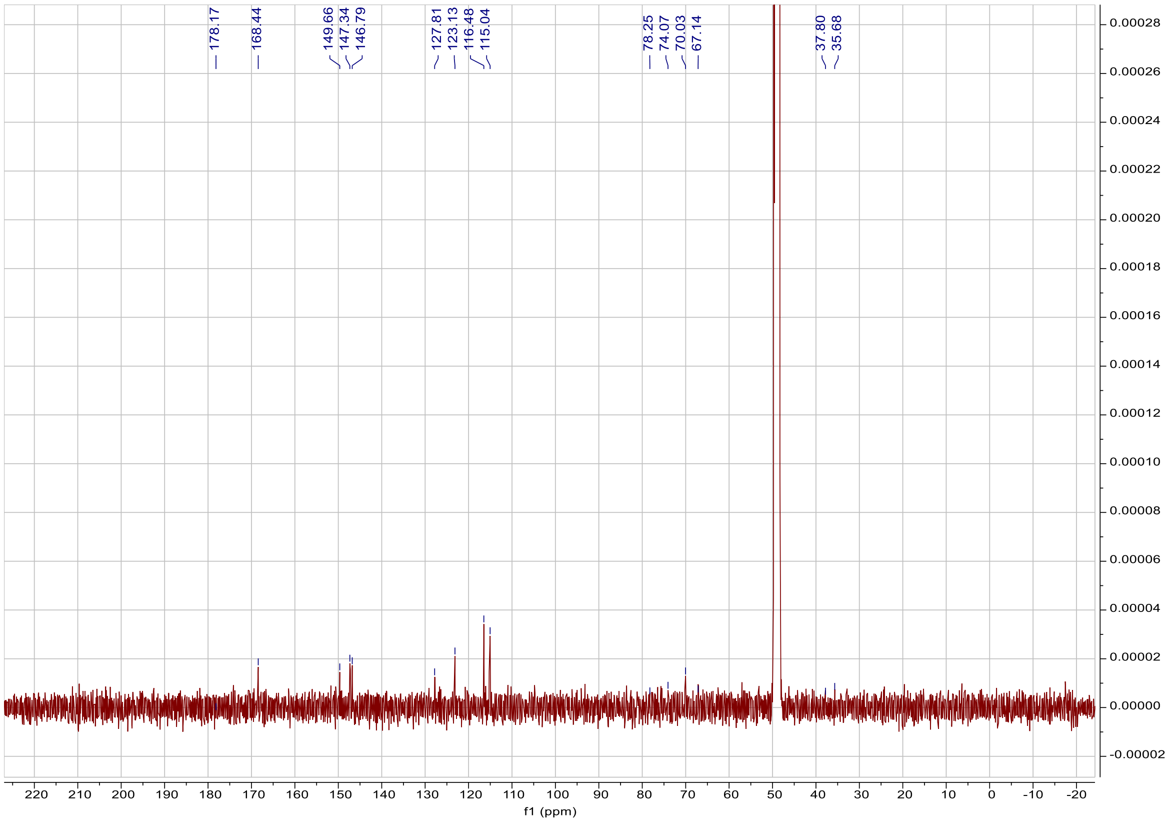

Figure S12. ^{13}C NMR (100 MHz, MeOH-d_4) spectrum of **5**

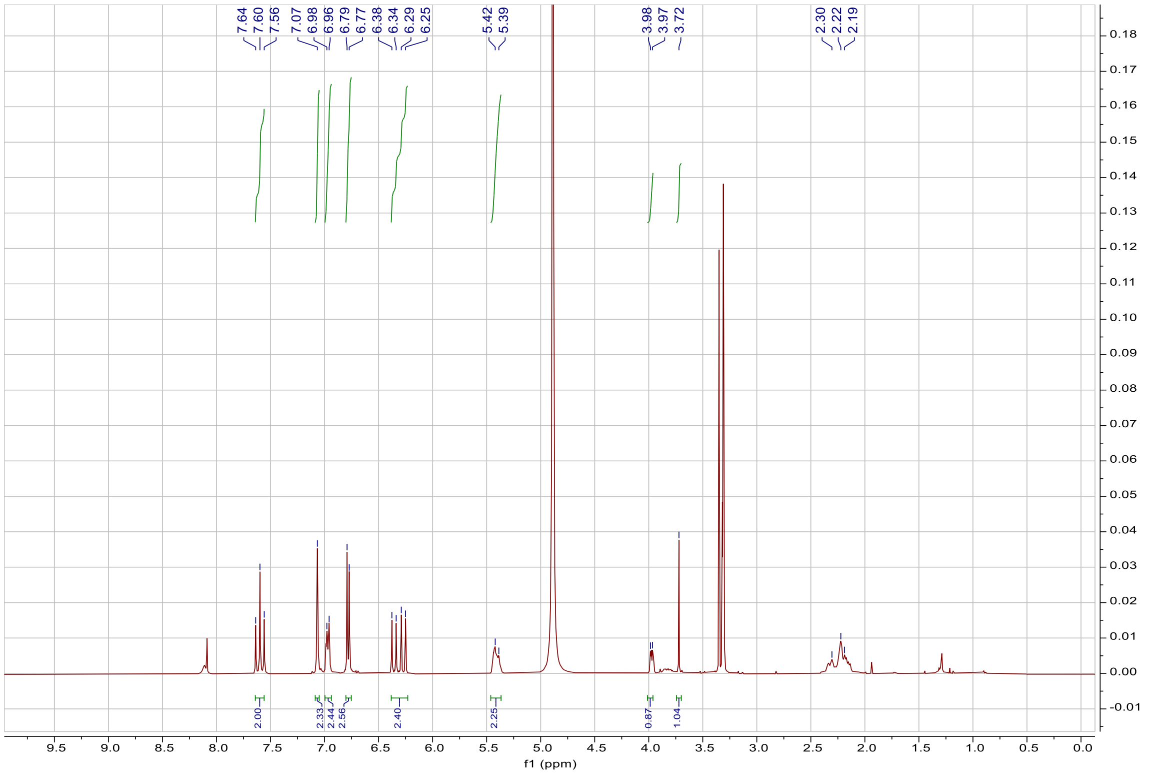

Figure S13. ^{1}H NMR (400 MHz, MeOH-d_4) spectrum of **6**

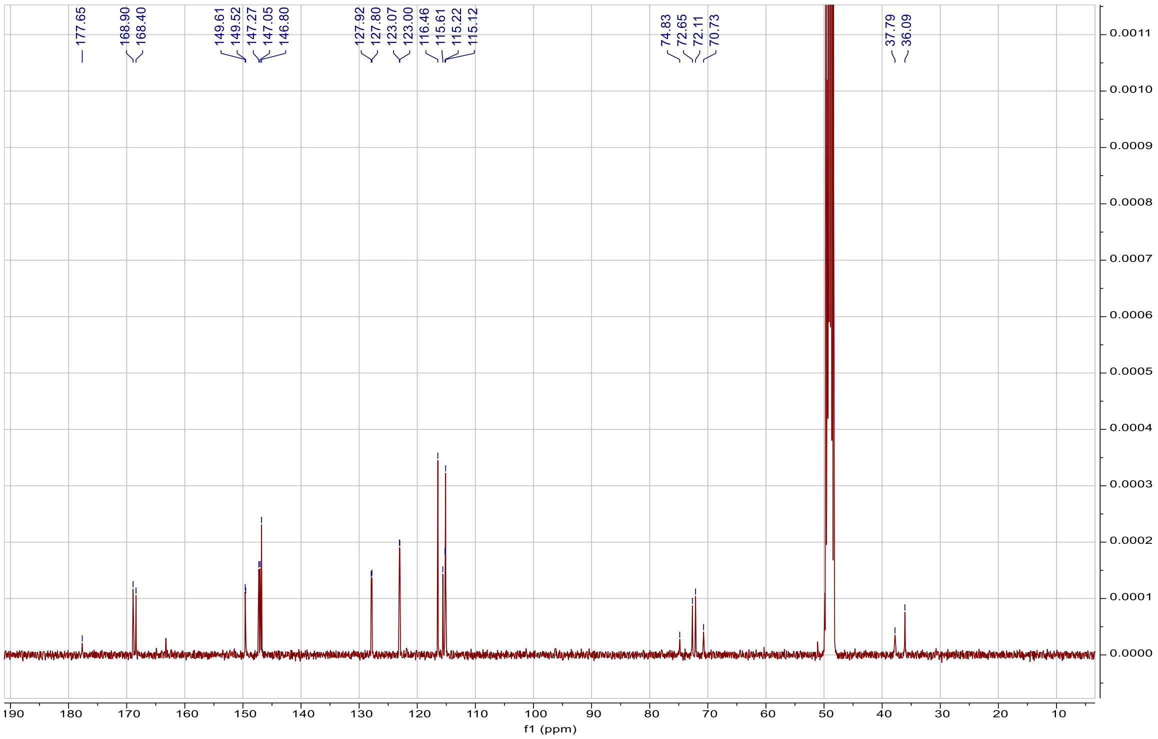

Figure S14. ^{13}C NMR (100 MHz, MeOH-d_4) spectrum of **6**

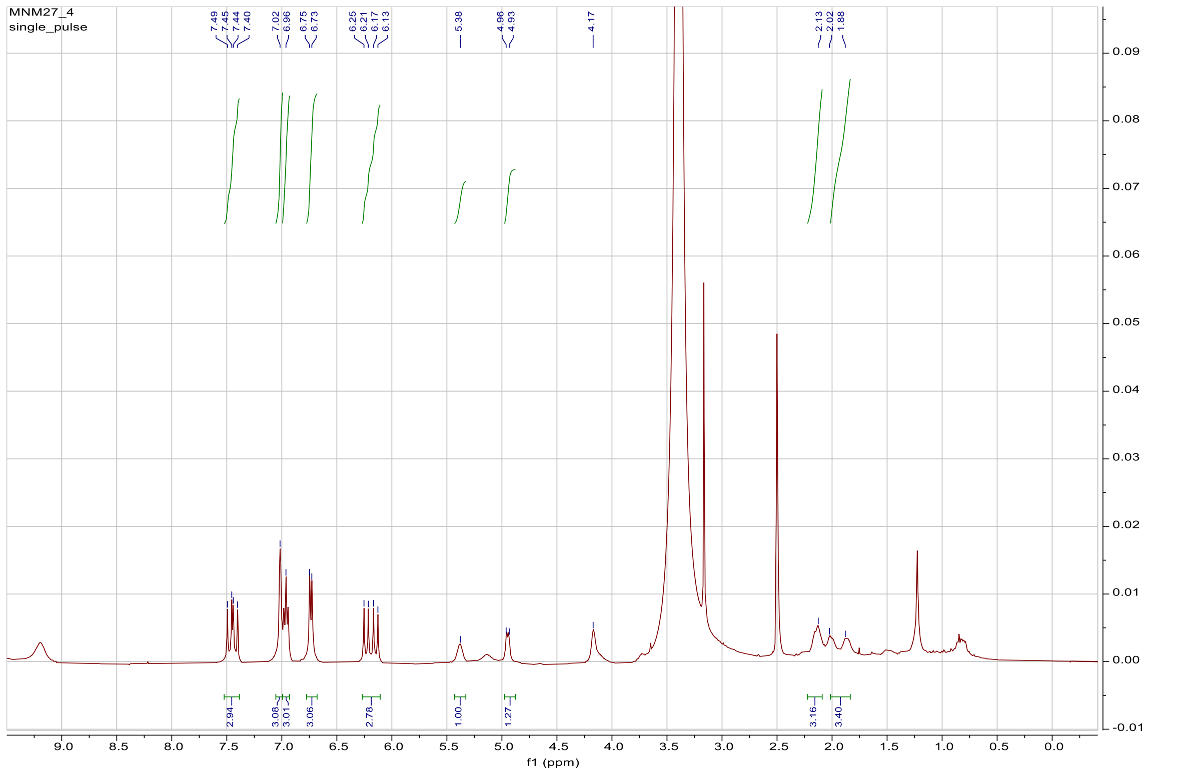

Figure S15. ^{1}H NMR (400 MHz, DMSO-d_6) spectrum of **7**

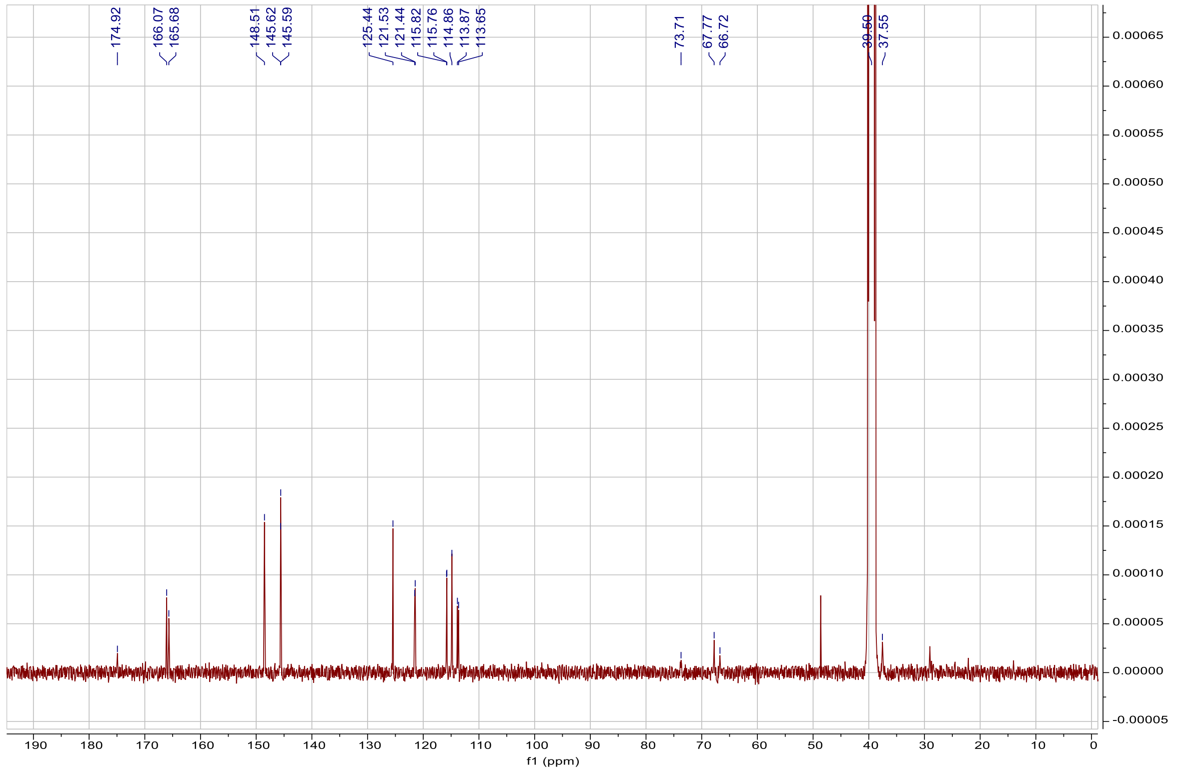

Figure S16. ^{13}C NMR (100 MHz, DMSO-d_6) spectrum of **7**

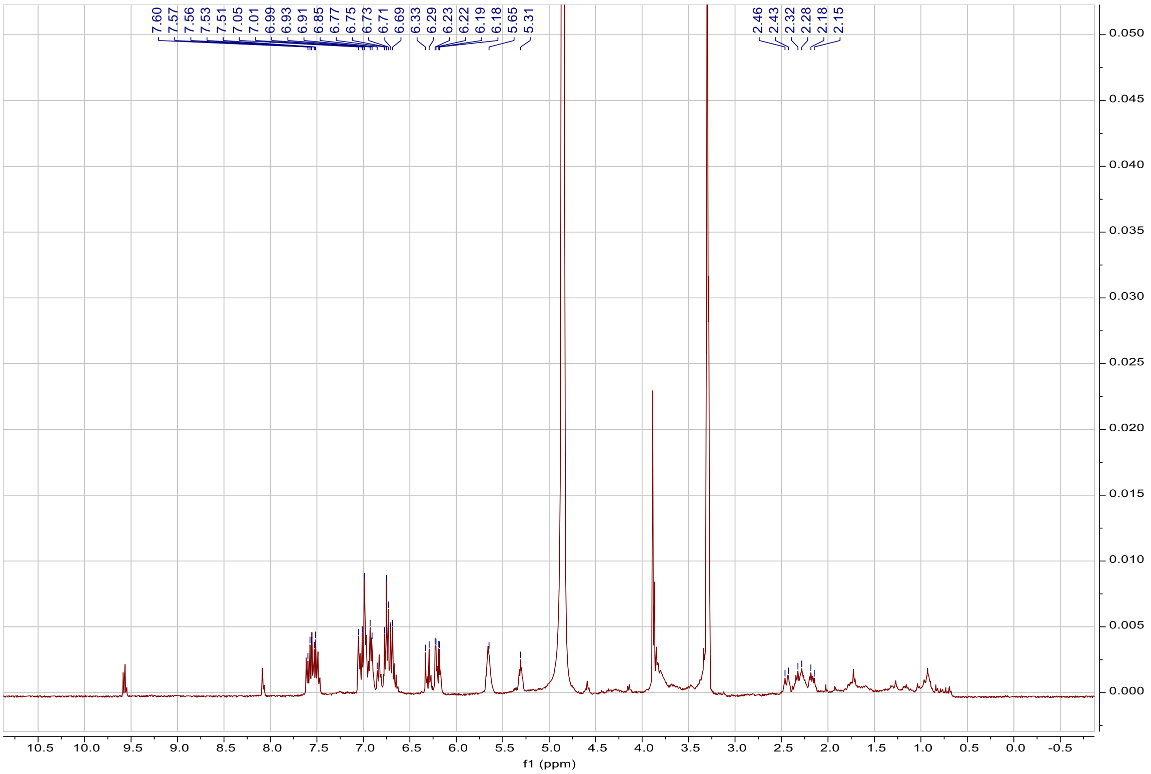

Figure S17. ^{1}H NMR (400 MHz, MeOH-d_4) spectrum of **8**

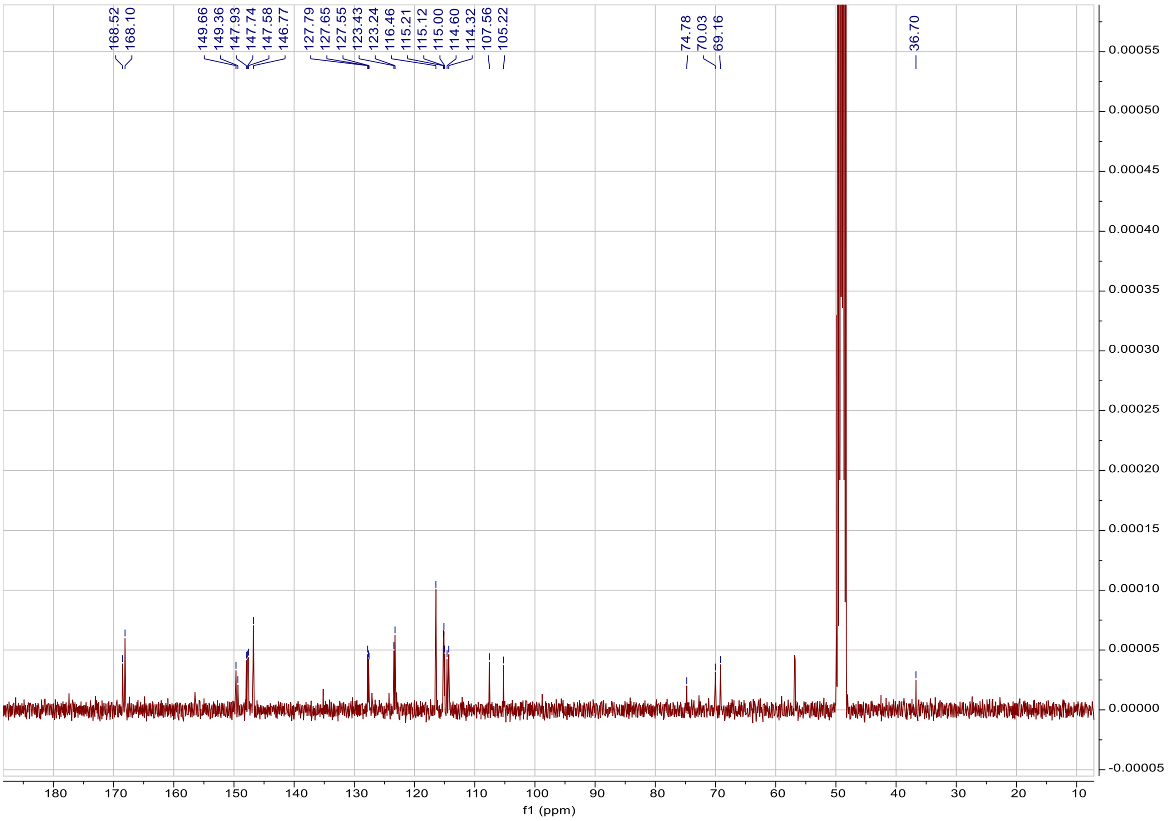

Figure S18. ^{13}C NMR (100 MHz, MeOH-d_4) spectrum of **8**

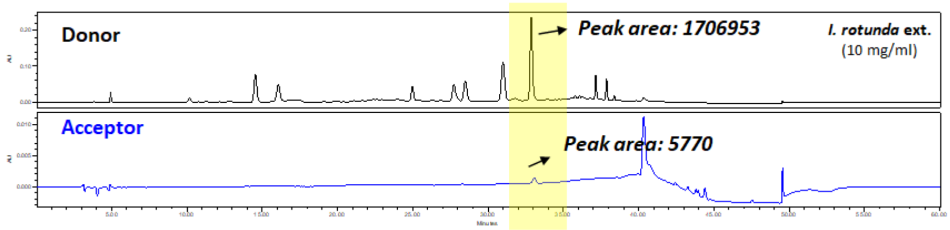

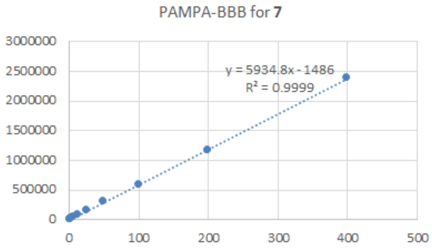

PAMPA-donor for **7**
Peak area: 1706953
X = $C_{D(t)}$ = (1706953+1486)/5934.8
= 287.86 (μg/ml)

PAMPA-acceptor area for **7**
Peak area: 5770
X = $C_{A(t)}$ = (5770+1486)/5934.8
= 6.62 (μg/ml)

A: 0.3 cm^2
t: 14400 (s)
$C_{D(t)}$: 0.28786 (mg/ml)
$C_{A(t)}$: 0.00662 (mg/ml)
V_D : 0.15 (ml)
V_A : 0.3 (ml)

$$C_{\mathrm{equilibrium}} = [C_{D(t)} \times V_D + C_{A(t)} \times V_A]/(V_D + V_A)$$

$C_{\mathrm{equilibrium}}$=0.100366667 (mg/ml)

$$P_e = \frac{-\ln\left[1 - C_{A(t)}/C_{\mathrm{equilibrium}}\right]}{A \times \left(\frac{1}{V_D} + \frac{1}{V_A}\right) \times t}$$

$$P_e = \frac{-\ln(0.934041847)}{43200} = \frac{0.068234038}{43200} = 1.57\text{x}10^{-6}$$

Permeability log P_e = log (1.57x10^{-6}) = -5.80

Compound **1**-**6** and **8** = acceptor area=0

$$P_e = \frac{-\ln(1)}{43200} = \frac{0}{43200}$$

not detected in acceptor or log P_e < −6.0 cross BBB potential = - PAMPA-BBB potential permeability based on Könczöl et al.

J. Pharm. Biomed. Anal. **2016**, *131*, 167-174.

Figure S19. PAMPA-BBB permeability test result for *I. rotunda* ext. (10 mg/ml) and detailed calculation procedure of permeability value for compound **7**

Figure S20. PAMPA-BBB permeability test results for compounds **1-8** (10 mM)

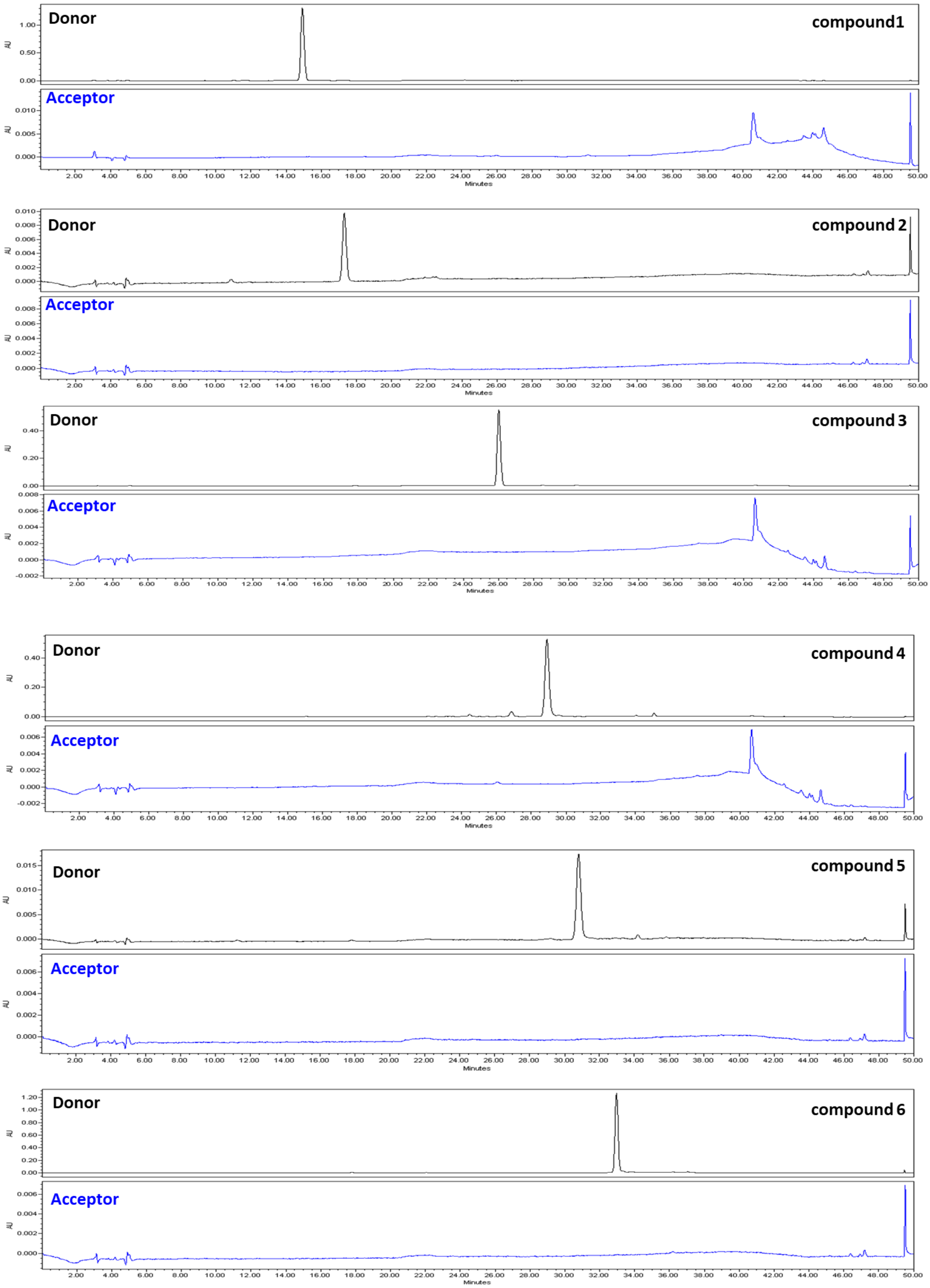

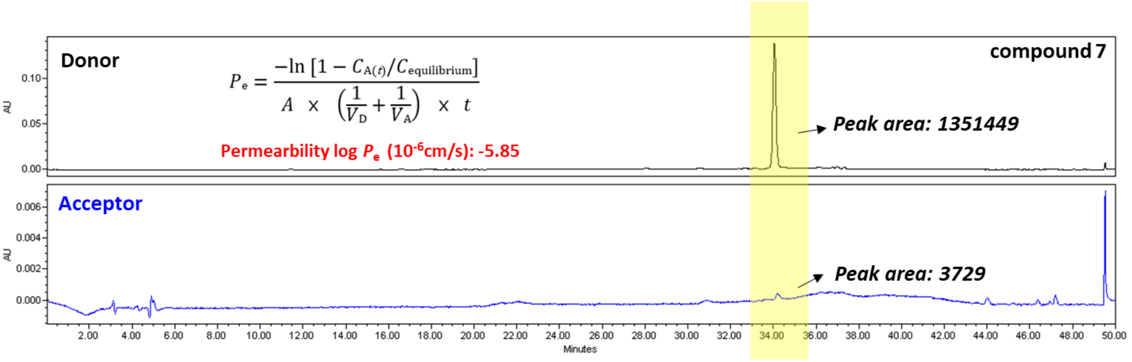
Donor
compound 7
$$P_e = \frac{-\ln[1 - C_{A(t)}/C_{equilibrium}]}{A \times \left(\frac{1}{V_D} + \frac{1}{V_A}\right) \times t}$$
Permearbility log P_e (10^{-6}cm/s): -5.85
Peak area: 1351449
Acceptor
Peak area: 3729
AU
Minutes

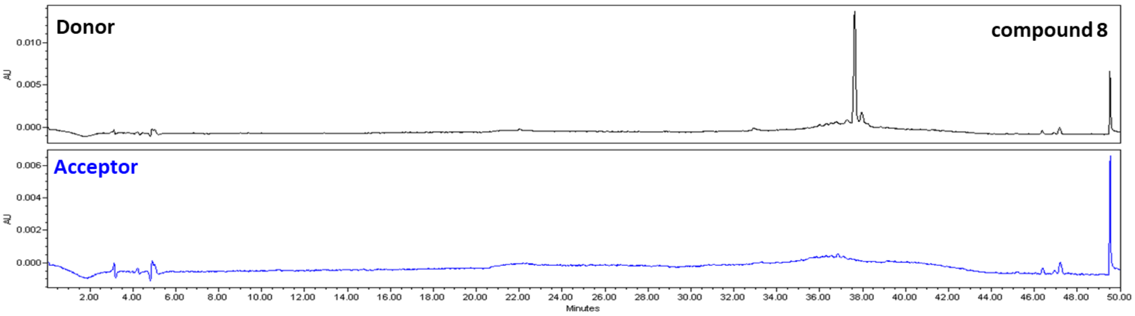
Donor
compound 8
Acceptor
AU
Minutes
